# Dealing with change in process choreographies: Design and implementation of propagation algorithms^☆^

**DOI:** 10.1016/j.is.2014.10.004

**Published:** 2015-04

**Authors:** Walid Fdhila, Conrad Indiono, Stefanie Rinderle-Ma, Manfred Reichert

**Affiliations:** a Faculty of Computer Science, University of Vienna, Austria; b Institute of Databases and Information Systems, University of Ulm, Germany

**Keywords:** Process-aware information system, Process choreography, Change propagation, Process change, Business collaboration

## Abstract

Enabling process changes constitutes a major challenge for any process-aware information system. This not only holds for processes running within a single enterprise, but also for collaborative scenarios involving distributed and autonomous partners. In particular, if one partner adapts its private process, the change might affect the processes of the other partners as well. Accordingly, it might have to be propagated to concerned partners in a transitive way. A fundamental challenge in this context is to find ways of propagating the changes in a decentralized manner. Existing approaches are limited with respect to the change operations considered as well as their dependency on a particular process specification language. This paper presents a generic change propagation approach that is based on the Refined Process Structure Tree, i.e., the approach is independent of a specific process specification language. Further, it considers a comprehensive set of change patterns. For all these change patterns, it is shown that the provided change propagation algorithms preserve consistency and compatibility of the process choreography. Finally, a proof-of-concept prototype of a change propagation framework for process choreographies is presented. Overall, comprehensive change support in process choreographies will foster the implementation and operational support of agile collaborative process scenarios.

## Highlights

• Enabling flexibility and change in process choreographies.
• Proposing change propagation algorithms for process choreographies.
• Handling transitive change propagation across several partners.
• Ensuring consistency and compatibility when propagating changes.
• First comprehensive proof-of-concept implementation of change propagation framework for process choreographies.

## 1 Introduction

The optimal design and implementation of their business processes is crucial for enterprises. This not only applies to internal business processes, but also to collaborative processes whose execution involves different partner enterprises. Examples include cross-organizational manufacturing [1] and tourism [2]. The system-based support of such collaborative processes is realized by *process choreographies* [3]. In particular, a *choreography model* describes the interactions between the partner processes through message exchanges. In a supply chain process, for example, the Supplier interacts with the Manufacturer and the Manufacturer with the Customer. The Customer, for example, may place an order with the Manufacturer by sending a corresponding order message.

In general, the interactions among the partners are visible to the outside and described by so called *public process models* (*public model* for short). In turn, the public models constitute views on the underlying internal partner processes, the so-called *private processes*. In particular, the models of the latter (i.e., private models) are not visible to the other partners due to confidentiality reasons. Altogether, a choreography model consists of the participating partners, a *global* view on all partner interactions, and the public as well as private models of the partners.

### 1.1 Research challenges

*Process change* has been identified as crucial in most application domains [4–6,58]. The demand for changing business processes arises due to various reasons such as the advent of new regulations or the emergence of new competitors at the market. Research on this topic has been extensive and led to flexible process management technology realized as mature commercial (e.g., AristaFlow^1^) and prototypical systems (e.g., CPEE^2^). So far, however, approaches dealing with process changes have focused on scenarios in which a business process is entirely run within a single enterprise. In turn, little attention has been paid to changes of process choreographies, even though the latter demand for agility and flexibility as well [7–9].

When applying changes to the processes supported by an information systems, in general, it must be ensured that neither structural nor behavioral soundness of the process is violated after the change [6]. For process choreographies, additional properties must be guaranteed due to the complexity introduced by the involvement of autonomous partners as well as the interactions between them. For example, assume that a particular partner applies a change to its private process. In addition to ensuring structural and behavioral soundness of this private process, it must be determined whether its change affects other partners in the choreography as well. Amongst others, this means that it must be checked whether the change of the private model affects the corresponding public model. In this case, it must be further ensured that the private model remains *consistent* with the public model. Note that this might require adaptations of the public model as well.

In turn, changing the public model of a particular partner might affect its interactions with other public models, e.g., when deleting an activity that sends a particular message another partner is waiting for. In order to ensure *compatibility* between the public models of the involved partners, therefore, one may have to *propagate* the changes from one partner and its public model to the other partners and their public models. After adapting the public models of the partners, in turn, the consistency with the underlying private models must be re-checked to ensure overall consistency. Note that change propagation cannot always be restricted to direct partners, but might spread *transitively* over the entire process choreography.

In general, propagating changes must not infringe the autonomy of the partners. In fact, adaptations becoming necessary to maintain the consistency and compatibility of the choreography should be suggested to partners, but the decision whether or not to adopt these adaptations must be left to them and may be subject to negotiations. In general, such negotiations can be costly and time-consuming, particularly in case of failure. This paper focuses on the fundamentals of change propagations in process choreographies whereas negotiation issues are discussed in [60,59]. Another challenge concerns the non-availability of information about the private processes of the partners. Hence, determining the adaptations required for the public and private models of the partners during change propagation is a difficult task.

Altogether, an approach enabling change propagation in process choreographies must tackle the following research challenges:

1. It must provide change propagation algorithms that ensure consistency and compatibility for all affected partners.
2. It must handle transitive change propagation across multiple partners.

In order to obtain an operational change propagation framework, we must further deal with implementation concepts required for realizing the change propagation algorithms for process choreographies.

### 1.2 Contribution

This paper provides an extended and revised version of the work we presented in [8]. First of all, [8] introduced fundamental notions as well as design decisions such as representing choreography processes as Refined Process Structure Trees (RPST) [12] and restricting the set of change operations to the insertion, replacement and deletion of process fragments (as described in [13]). This paper adopts these design decisions. Further on, [8] addressed Research Challenges 1 and 2 by providing propagation algorithms for change operations REPLACE and UPDATE, whereas other change operations were not considered. Finally, [8] discussed how the propagation algorithms ensure consistency and compatibility of the choreography model and highlighted the problem of transitive change propagation.

Compared to [8], this paper provides significant revisions and extensions of the results related to Research Challenges 1 and 2. This includes (i) a fundamental revision of the previous definitions using the mapping functions between the different choreography models and – in the sequel – the propagation algorithms; (ii) the propagation algorithms for additional change operations (i.e., Insert and Delete), (iii) extensive illustrations of the algorithms, (iv) a revision of the Replace algorithm, (v) an extended discussion on transitivity when propagating changes in process choreographies, and (vi) an extension of the formal evaluation of consistency and compatibility in the context of respective change. Furthermore, this paper provides novel results regarding the technical evaluation of our approach. We propose an architecture for implementing a sophisticated change propagation framework for process choreographies. This architecture consists of three layers for defining, executing and changing processes. The core component of the change layer, which is realized as a proof-of-concept prototype, is the C^3^Editor. The latter allows for the import of private, public and choreography models from tools such as Signavio and jBPM. The C^3^Pro Editor visualizes the different models and enables the definition and application of changes to the private models. Furthermore, it determines and visualizes the partners affected by a change and the updates required for change propagation. To the best of our knowledge, this is the first prototype enabling change and change propagation in process choreographies. This paper is organized as follows: Section 2 introduces a motivating example, followed by fundamental definitions in Section 3. Section 4 then presents the change propagation algorithms we developed. Section 5 discusses the handling of transitivity when propagating changes in process choreographies. Our approach is evaluated in Section 6 regarding the consistency and compatibility of the choreography after change propagation. Section 7 provides the details on the architecture and proof-of-concept implementation. In Section 8, we discuss related work. Section 9 summarizes the paper.

## 2 Running example and model representation

From the perspective of a single partner, three different, but overlapping viewpoints form a collaboration: the private model, public model, and choreography model [16].

• The *private model* describes the internal business logic as well as the message exchanges this partner is engaged in; i.e., the private model corresponds to the executable process of this partner. In general, the internal logic is not visible to other partners.
• The *public model* sketches the message exchanges from the perspective of this single partner as well as their sequencing; i.e., it represents an abstraction of the private activities corresponding to the private model. Compared to the public model, the private model contains the business process logic not visible to the other partners.
• The *choreography model* provides a global view on the interactions of a collaboration; i.e., it captures all interactions among the partners as well as the dependencies between these interactions.

### 2.1 Running example

**Fig. 1:**
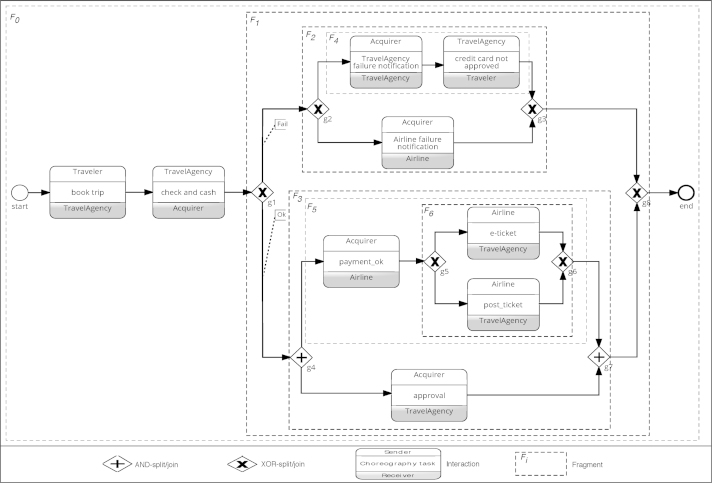
Choreography model: simplified book trip process [2].

We illustrate change propagation issues along the *booking trip* choreography example depicted in Fig. 1. This example is part of the choreography model described in [2]. It has been modeled using the choreography diagram elements of BPMN 2.0 and the Signavio tool [14]. The example describes a collaboration among four partners, i.e., *traveler*, *travel agency*, *acquirer*, and *airline*. The *traveler* sends booking information to the *travel agency* that, in turn, contacts the *acquirer* to request a credit check. If the *traveler* does not have enough credit, failure notifications are sent to the *travel agency* and *airline*, which inform the *traveler* about the reservation failure and purchase cancellation, respectively. Otherwise, an approval is sent to the *travel agency* and the *airline* is triggered to send the ticket and the purchase confirmation.

**Fig. 2:**
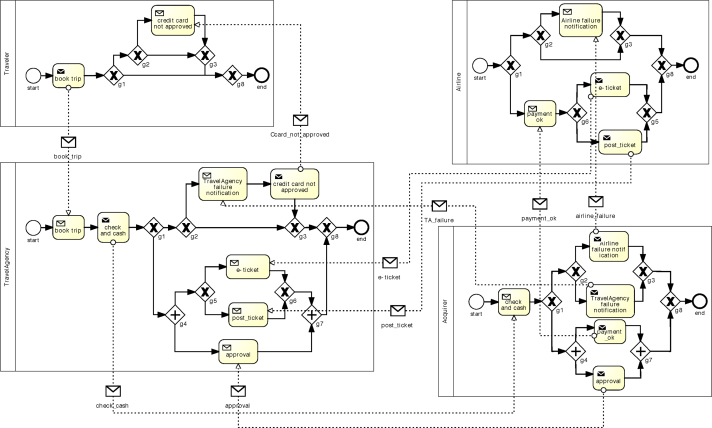
Book trip process: public models.

**Fig. 3:**
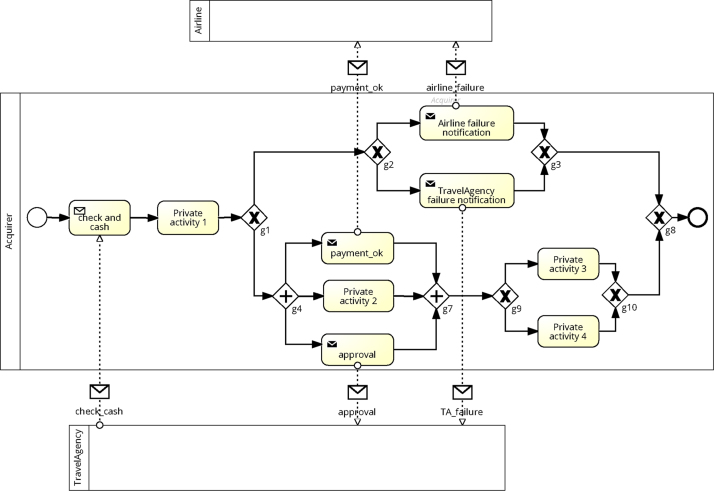
Book trip process: acquirer private model.

Fig. 2 depicts a BPMN collaboration diagram listing the public models of all partners involved in the choreography. Each public model includes the interactions the corresponding partner is involved in as well as the control flow between them. Note that Fig. 2 does not show the private models of the partners, which contain their internal activities (cf. Fig. 3). Finally, merged together, the public models lead to the choreography model.

As motivated, in many application scenarios, the partners of a collaboration should be allowed to change their private processes. The specific challenge compared to local changes of a single process is to *propagate* change effects from one partner to the others [8,9] if required. For example, the TravelAgency might want to send a questionnaire about customer satisfaction to the Traveler after booking the ticket. This could be accomplished by inserting corresponding activities into the private model of the TravelAgency; e.g., DevelopQuestionnaire, which constitutes an internal activity not visible to other partners, and SendQuestionnaire, which constitutes the public activity to be added as well. Furthermore, a respective *change request* needs to be sent to the Traveler who should be able to receive the corresponding message and respond to it.

In general, change propagation in process choreographies might become quite complex [8]. Consider the above example and assume that it is not the TravelAgency which initiates the collection of customer feedback, but the Airline through the Acquirer and TravelAgency. In this case, the initial change will cause *transitive* effects across multiple partners. To overcome this problem, the effects of this local change in the private model of one partner need to be propagated to the concerned partners. As a consequence, the interactions must be restructured accordingly.

### 2.2 Model representation

As a prerequisite for precisely defining the notions of private, public and choreography model, we need to be able to represent the control-flow relations between activities and interactions. With the *Refined Process Structure Tree* (*RPST*) [12], this paper adopts a structured representation for this. An RPST model corresponds to a decomposition of a process model into a set of single-entry, single-exit (SESE) fragments. Thereby, each node of an RPST represents a SESE fragment of the underlying process model. Consequently, the root node corresponds to the entire process model, whereas the child nodes of a node N correspond to the SESE fragments directly contained under N; i.e., the RPST parent–child relation corresponds to the containment relation between SESE fragments. As a key characteristic, the RPST can be constructed for any process model captured in a graph-oriented notation [17].

We choose the RPST for various reasons. Besides being generic and language-independent, the RPST is indeed a structured tree representation of a given model. Note that *structured process models* are close to BPEL and are simpler to analyze and easier to comprehend than unstructured models. However, recent work has shown that most unstructured process models can be automatically translated into structured ones [18]. Additionally, computing and propagating changes for unstructured processes is rather complex and might violate the soundness of the choreography. Transforming unstructured processes into structured ones, therefore, eases the propagation of changes and ensures a more sound propagation. Furthermore, using tree structures instead of usual graph representations significantly reduces the complexity for calculating the impacts of a change (e.g. parsing, identifying fragments). Indeed, high-level change operations (cf. Section 3) refer to entire process fragments (i.e., sets of activities and gateways) instead of single nodes. As process models are block structured in RPSTs, in turn, this makes it easier to identify the fragments to be modified in the processes of the partners involved in a change. Finally, in [12] it was proven that the translation of the process models to block-structured languages (e.g. BPEL) becomes easier through their decomposition into RPST. The transformation of graph models to RPST is linear, idempotent and modular [12].

**Fig. 4:**
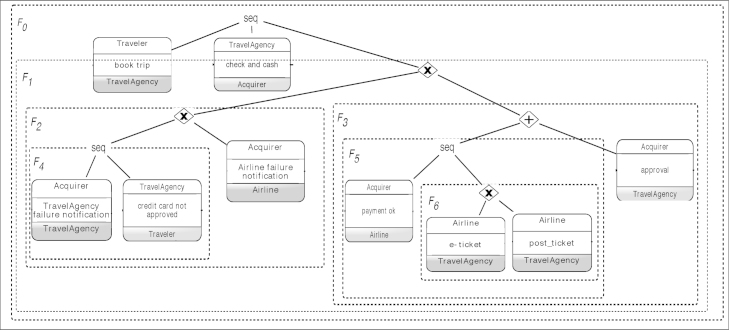
Process structure tree of the book trip operation.

Fig. 4 depicts the tree model of the choreography scenario from Fig. 1. In essence, the interaction nodes of the original graph are mapped to leaves in the tree model and represent the *Trivial nodes*, whereas the control nodes (i.e., sequence (SEQ), choice (CHC), parallel (PAR), or loop (RPT)) are mapped to internal nodes (for more details see [12]).

## 3 Fundamental definitions

This section introduces the main definitions used throughout the paper. Section 3.1 provides the formal definitions related to the various models a choreography is composed of. In turn, Section 3.2 presents basic definitions related to change, change propagation, and change operations.

### 3.1 Process choreography

A choreography includes three types of models: (i) the private model representing the executable process and including private activities as well as interactions with other partners, (ii) the public model (also called the interface of the process) highlighting solely the interactions of a given partner, and (iii) the choreography model giving a global view on the interactions between all partners. In the following we sketch the corresponding definitions.

Definition 1 **(Structured) Private Model**

A private model $\pi_{p}$ of partner *p* corresponds to a tree with the following structure^3^:

Process::=PNodePNode::=Activity|ControlNode|EventActivity::=PrivateActivity|InteractionActivityInteractionActivity::=Send(Message,Receiver)|Receive(Message,Sender)ControlNode::=SEQ({PNode})|CHC({PNode})|PAR({PNode})|RPT(PNode)Event::=Start|End

SEQ corresponds to a sequence of fragments, CHC to a choice between two or more fragments, PAR to a parallel execution of several fragments, and RPT to an iteration over a fragment.

Example 1 In the private process model depicted in Fig. 3, fragment F is represented as follows:

SEQ(PAR(Send(payment_ok,airline),pr_activ2,Send(approval,travelAge_ncy)),XOR(pr_activ3,pr_activ4))

Definition 2 **(Structured) Public Model**

The public model *l*_*p*_ of a partner *p* reflects the external behavior of *p*; i.e., it includes the interactions with other partners as well as the constraints between them from the viewpoint of *p*:

LocalModel::=LNodeLNode::=InteractionActivity|ControlNode|EventInteractionActivity::=Send(Message,Receiver)|Receive(Message,Sender)ControlNode::=SEQ({LNode})|CHC({LNode})|PAR({LNode})|RPT(LNode)Event::=Start|End

Fig. 2 represents a collaboration diagram that illustrates the different public models of the book trip choreography example. Note that each panel defines the public model of one single partner.

Definition 3 **(Structured) Choreography Model**

A global choreography model G represents a global view on the interactions between collaborating partners.

ChoreographyModel::=CNodeCNode::=I(Sender,Receiver,Message)|ControlNode|EventControlNode::=SEQ({CNode})|CHC({CNode})|PAR({CNode})|RPT(CNode)Event::=Start|End

*I* corresponds to an interaction between partners *Source* and *Destination* (i.e., the exchange of message *Message*).

An example of a choreography model is illustrated in Fig. 1. We define a *fragment* F as a non-empty subtree of a private model, public model or choreography model with single entry and single exit edge (SESE). Regarding Definitions 1–3, a tree model fragment is represented by elements *PNode*, *LNode* and *CNode*, respectively. Next, we define a choreography as the aggregation of all elements necessary for ensuring a sound collaboration between the participating partners.

Definition 4 **Choreography**

We define a choreography C as a tuple (G, P, Π, L, *ψ*, *φ*, *ξ*) where,

• G is the choreography model (cf. Definition 3).
• P is the set of all participating partners.
• $\Pi ={\left\{\pi_{p}\right\}}_{p\in P}$ is the set of all private models (cf. Definition 3).
• $L={\left\{l_{p}\right\}}_{p\in P}$ is the set of all public models (cf. Definition 2).
• $\psi ={\left\{\psi_{p}:l_{p}↔\pi_{p}\right\}}_{p\in P}$ is a partial mapping function between nodes of the public and private models.
• *φ*: l↔l′ is a partial mapping function between nodes of different public models.
• ξ:G↔l is a partial mapping function between nodes of the choreography model and the public models.

Functions *ψ* and *φ* can be used to check the consistency between public and private models (i.e., each private model must be consistent with the respective public model) as well as the compatibility between public models.

### 3.2 Change and change propagation

**Fig. 5:**
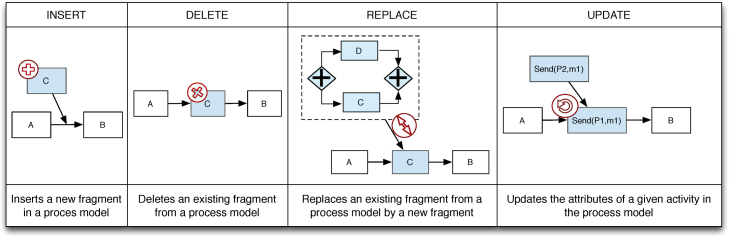
Change patterns.

In order to represent changes of a choreography, we consider four basic change patterns: REPLACE, DELETE, INSERT, and UPDATE (cf. Fig. 5).

Definition 5 **Change Patterns**

ChangePattern:≔REPLACE(oldFragment,newFragment)|DELETE(fragment)|INSERT(fragment,how,pred,succ)|UPDATE(activity,attribute,newValue)how::=Parallel|Choice|Sequenceattribute::=partner|role|Input|Output

REPLACE allows replacing an existing fragment with a new one. DELETE removes an existing fragment, whereas INSERT adds a new fragment to the process model between a predecessor node *pred* and a successor node *succ*. Finally, UPDATE allows modifying an attribute of a single activity as illustrated in Fig. 5. Note that more complex changes can be expressed by combining these four patterns. Change patterns are defined as follows:

Definition 6 **Change Operation**

A change operation is a tuple (*δ*,*σ*) where *σ* is either the private, public or choreography model to be changed, and *δ*:σ↦σ′ corresponds to the change that transforms *σ* into σ′.

Example 2 Consider Fig. 3: $\mathrm{DELETE}(\mathrm{check}\_\mathrm{and}\_\mathrm{cash},\pi_{\mathrm{Acquirer}})$ deletes the activity check_and_cash from the private model of the Acquirer.

Definition 7 **Abstraction Function**

An abstraction function ${\mathrm{abstr}}_{\lambda }:\sigma \mapsto \sigma \prime$ is a projection of a model *σ* according to criterion *λ*. The following holds:

• ∀n∈σ with *n* satisfies *λ*, ⟹n∈σ′ (n refers to node).
• ∀n,n′∈σ with n,n′ satisfying *λ* and *n* precedes n′ in σ,⟹n,n′∈σ′∧n precedes n′ in σ′.

Function ${\mathrm{abstr}}_{\lambda }(\sigma )$ transforms a source model *σ* into a target model σ′ that solely contains activities satisfying *λ*; e.g., a public model corresponds to an abstraction of a private model with respect to interactionactivities (cf. Definitions 1 and 2). The abstraction of a private model may further contain structures not contributing to process execution (e.g., “empty” branches in a parallel branching). In this case, refactorings may be applied [20]. Next, we assume that λ=p′ refers to the interactions with p′. Hence, ${\mathrm{abstr}}_{p\prime }(l_{p})$ corresponds to the abstraction of *l*_*p*_ according to the interactions of *p* with p′. As result, we obtain a view on all interactions *p* has with p′. The abstraction function allows calculating the propagation effects; e.g., by identifying the effects a change of a private model has on its corresponding public model.

Example 3 The result of abstracting fragment F (cf. Example 1) according to its interactions is as follows:

PAR(Send(payment_ok,airline),Send(approval,travelAge_ncy))

Definition 8 **Complement Function**

Assume that a∈p corresponds to an interaction activity with a partner p′. Then: The complement of *a*, which is denoted as $\overset{¯}{a}\in p\prime$, corresponds to the opposite of *a*, i.e.,

• $\overset{¯}{\mathrm{send}(\mathrm{message},p\prime )}=\mathrm{receive}(\mathrm{message},p)$.
• $\overset{¯}{\mathrm{receive}(\mathrm{message},p\prime )}=\mathrm{send}(\mathrm{message},p)$.

If F corresponds to a fragment solely consisting of interaction activities, $\overset{¯}{F}$ corresponds to a fragment having the same structure as F and replacing each activity of F with its complement. This function is required to maintain the compatibility between process partners when propagating changes.

Given an arbitrary set of nodes of a model *σ*, we define *α* as the function returning the smallest fragment in *σ* containing all these nodes. This function allows keeping the effects of a change as local as possible.

Definition 9 **Smallest fragment *α***

Let *σ* be a model and S be a set of corresponding nodes. Then: $\alpha_{\sigma }(S)$ returns the smallest fragment in *σ* containing all nodes from S. Formally: $\alpha_{\sigma }(S)={\mathrm{argmin}}_{\mathrm{size}(F)}\{F\in \sigma |\forall n\in S,n\in F\}$.

Example 4 In Fig. 1, $\alpha_{G}(\{\mathrm{payment}\_\mathrm{ok},\mathrm{approval}\})=F_{3}$ holds.

Usually, determining the changes to be propagated to the partner processes requires knowledge about the activities executed before or after the changed fragment. In this context, the following definitions are useful.

Definition 10 **Preset (Postset)**

The *preset* (*postset*) of a node *n* in model *σ* corresponds to the set of nodes in *σ* that can be executed directly before (after) *n*. Formally:

• preset(n,σ)={n′∈σ|∃SEQ(n′,n)∈σ}
• postset(n,σ)={n′∈σ|∃SEQ(n,n′)∈σ}

We further define the *preset* (*postset*) of a fragment F in a given model *σ* as the fragment of *σ* that can be executed directly before (after) F.

Example 5 Consider Fig. 1. We obtain preset(check_and_cash,G)={book_trip} and $\mathrm{postset}(g_{1},G)=\{g_{2},g_{4}\}$.

Definition 11 **$T\_{\mathrm{preset}}_{\lambda }$ ($T\_{\mathrm{postset}}_{\lambda }$)**

In a model *σ*, the transitive preset (postset) of a node *n*, according to criterion *λ*, represents the set of nodes that satisfy *λ* and can be executed directly before (after) *n*.

• $T\_{\mathrm{preset}}_{\lambda }(n,\sigma )=\mathrm{preset}(n,{\mathrm{abstr}}_{\lambda }(\sigma ))$
• $T\_{\mathrm{postset}}_{\lambda }(n,\sigma )=\mathrm{postset}(n,{\mathrm{abstr}}_{\lambda }(\sigma ))$

The transitive preset (postset) $T\_{\mathrm{preset}}_{\lambda }$ ($T\_{\mathrm{postset}}_{\lambda }$) of fragment F, according to criterion *λ*, represents the smallest fragment F′ of *σ* that contains all nodes satisfying *λ* and being executable directly before (after) F.

Example 6 Consider Fig. 1. We obtain

• $T\_{\mathrm{preset}}_{\mathrm{Acquirer}}(\mathrm{check}\_\mathrm{and}\_\mathrm{cash},G)=\{\mathrm{start}\}$, *and*
• $T\_{\mathrm{postset}}_{\mathrm{Traveler}}(g_{2},G)=\{\mathrm{credit}\_\mathrm{card}\_\mathrm{not}\_\mathrm{approved}\}$.

## 4 Change propagation

**Fig. 6:**
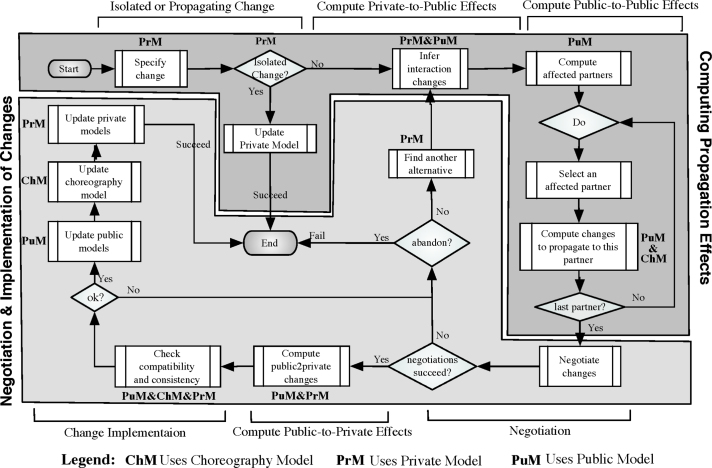
Change propagation—major steps (adopted from [8]).

Our goal is to enable change propagation in choreographies with multiple interacting partner processes. Our approach is based on six major steps: (i) checking whether a change needs to be propagated or is isolated (i.e., the change is local), (ii) computing the private-to-public effects; i.e., propagating changes from the private model of the change initiator to its public model, (iii) computing the public-to-public effects; i.e., propagating changes to the partners involved, (iv) negotiating the changes with the concerned partners, (v) computing public-to-private effects (if negotiations have succeeded); i.e., each partner calculates internally the effects of the public changes on its private model, and finally (vi) checking the compatibility and consistency of the choreography and implementing the changes. Fig. 6 details these steps and outlines the different actions required to achieve a sound propagation.

When applying a change operation to a partner׳s private model, we extract all interaction activities concerned by the change–interaction activities are message exchanges with other partners (i.e., sending and receiving messages). If the list is empty (i.e., the change is restricted to the internal behavior), the other partners are not affected by the change. Hence, there is no need for any new agreement on the global choreography. Otherwise, the list of affected interactions is analyzed to identify all partners involved. Then, for each of these partners, a relative change computation is accomplished to determine the changes to be propagated. The latter are computed according to the change operation type. Then, a negotiation phase is launched with each affected partner. If all negotiations succeed, we apply consistency and compatibility checks to ensure the soundness of the obtained models. In turn, if these models are sound, we update the public models affected by the change as well as the choreography model and, if necessary, adapt concerned private models to their new public models. If negotiations do not succeed, either the change is canceled or it is tried to circumvent those partners with whom negotiations failed in the past. Note that this propagation strongly depends on the change pattern applied (i.e., INSERT, DELETE, REPLACE, or UPDATE). We sketch the different algorithms needed for propagating changes depending on the change pattern used. The propagation of change pattern UPDATE is not considered in this paper, but can be found in [8]. According to Fig. 6, the focus is on determining the public propagation effects of a single change (i.e., the parts in dark gray). In particular, we want to identify whether or not a change is isolated, and compute private-to-public and public-to-public effects. Negotiation and computing public-to-private-effects (i.e., effects of changing a partner׳s public model on its private model) are out of the scope of this paper

### 4.1 Propagation of fragment insertions

**Fig. 7:**
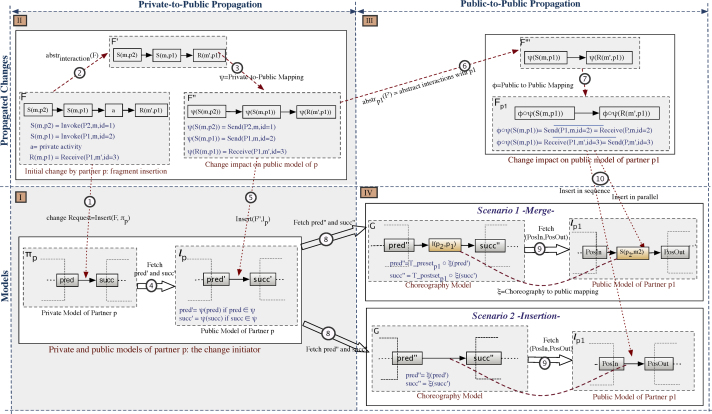
Major steps for propagating an insert operation.

The INSERT pattern is used to add a new fragment F to the private model *π*_*p*_ of a partner *p* between two consecutive nodes *pred* and succ. In the following, we use the example depicted in Fig. 7 to explain and illustrate the main propagation steps for propagating fragment insertions. Given a change operation *δ* of type INSERT applied to a partner׳s private model *π*_*p*_, and F being the fragment to be inserted in *π*_*p*_ between two nodes *pred* and *succ* (cf. Step 1 in Fig. 7), the ripple effects of *δ* can be computed asfollows:

1. *Isolated or propagating changes*: We first check whether F contains additional interactions or solely private activities by abstracting F with respect to interactions (cf. Step 2 in Fig. 7). If $F\prime ={\mathrm{abstr}}_{\mathrm{interaction}}(F)$ is not empty (i.e., F contains at least one node), new interactions have been added and a change propagation becomes necessary. Otherwise, the change is considered as isolated; i.e., no propagation is needed.
2. *Private-to-Public effects*: In order to compute the impacts on the public model of the change initiator, we proceed as follows:
  • If F′ is not empty, we calculate the corresponding fragment to be added to the public model *l*_*p*_ of change initiator *p*. The latter is the partner that initiated the change propagation. For this purpose, we use private-to-public mapping function *ψ* that transforms the elements of F′ into elements of *l*_*p*_. Note that this will be crucial if the private and public models are defined in terms of different modeling languages; e.g., in Fig. 7 the elements of *π*_*p*_ and F might be defined with BPEL, whereas the ones of F″ and *l*_*p*_ are defined in BPMN (cf. Step 3 in Fig. 7).
  • When inserting F between *pred* and *succ* in *π*_*p*_, this results in an insertion of F″ in *l*_*p*_. To maintain the consistency between *π*_*p*_ and *l*_*p*_, F″ should maintain the precedence relationship with *pred* and *succ*. Since *pred* and *succ* in *π*_*p*_ may be private activities without corresponding elements in *l*_*p*_, however, it becomes challenging to identify the insertion positions pred′ and succ′ of F″ in *l*_*p*_ (cf. Step 4 in Fig. 7). Therefore, we first check whether *pred* and *succ* constitute interaction activities or have corresponding elements in *l*_*p*_ (ψ(pred)≠∅). In this case, we consider the corresponding positions in *l*_*p*_; i.e., pred′=ψ(pred) and succ′=ψ(succ) respectively. Otherwise, we look at the elements of *π*_*p*_ having corresponding elements in *l*_*p*_ and directly preceding *pred* (i.e., $T\_{\mathrm{preset}}_{\mathrm{interaction}}(\mathrm{pred})$) and following *succ* (i.e., $T\_{\mathrm{postset}}_{\mathrm{interaction}}(\mathrm{succ})$). Then, F″ is inserted between the corresponding elements in *l*_*p*_ as follows (cf. Step 5 in Fig. 7):
    ○ $\mathrm{pred}\prime =\psi ○T\_{\mathrm{preset}}_{\mathrm{interaction}}(\mathrm{pred})$
    ○ $\mathrm{succ}\prime =\psi ○T\_{\mathrm{postset}}_{\mathrm{interaction}}\mathrm{succ})$
3. *Public-to-Public effects*: In order to calculate the change impacts on the other partners, we proceed as follows:
  • We analyze F″ in respect to the list of partners involved in the change. For each of these partners, we identify the interactions this partner is involved in. Given a partner *p*_1_, this induces the calculation of ${\mathrm{abstr}}_{p_{1}}(F\prime \prime )$ (cf. Step 6 in Fig. 7). In turn, abstraction function ${\mathrm{abstr}}_{p_{1}}$ returns a connected component including all interactions with *p*_1_; i.e., F‴.
  • F‴ represents the fragment to be inserted into the public model $l_{p_{1}}$ of *p*_1_. To preserve the compatibility of the collaboration, however, we must calculate the complement of F‴ (i.e., $F_{p_{1}}=\overset{¯}{F\prime \prime \prime }$) and update the public-to-public mapping function *φ* (cf. Step 7 in Fig. 7). The latter maintains the correlation between nodes of different public models.
  • Given the fragment F″ to be inserted between pred′ and succ′ in *l*_*p*_ (cf. Step 5 in Fig. 7), how can we identify the insertion positions of the corresponding fragment $F_{p_{1}}$ in $l_{p_{1}}$; i.e., *PosIn* and *PosOut* (cf. Step 10 in Fig. 7). This becomes challenging if pred′ and succ′ of *l*_*p*_ have no corresponding elements in $l_{p_{1}}$, or *p* has no interactions with *p*_1_. Utilizing the choreography model G then becomes primordial since it provides a global view on the interactions of all partners. Further, it contributes to identify the relationships between the elements pred′ and succ′ of *p*, and the interaction activities of *p*_1_. The problem is shifted to finding the corresponding elements of pred′ and succ′ in G (i.e., ξ(pred′) and ξ(succ′) respectively), using the public-to-choreography mapping *ξ*. Then, we analyze the interactions in G, *p*_1_ is involved in, and which precede ξ(pred′) and follow ξ(succ′); i.e., $\mathrm{pred}\prime \prime =T\_{\mathrm{preset}}_{p_{1}}(\xi (\mathrm{pred}\prime ))$ and $\mathrm{succ}\prime \prime =T\_{\mathrm{postset}}_{p_{1}}(\xi (\mathrm{succ}\prime ))$ respectively. Again, using the public-to-choreography mapping $\xi :G\to l_{p1}$, we identify the insertion positions in $l_{p_{1}}$ with PosIn=ξ(pred″) and PosOut=ξ(succ″). We distinguish two possible scenarios when inserting $F_{p_{1}}$:
    ○ Scenario 1: There exist no interaction activities between PosIn and PosOut in $l_{p_{1}}$. In this case, $F_{p_{1}}$ should simply be inserted between *PosIn* and *PosOut*.
    ○ Scenario 2: There exists a set *S* of interaction activities *PosIn* and *PosOut* in $l_{p_{1}}$. In this case, $F_{p_{1}}$ is merged with all elements of *S*.

### 4.2 Propagation of fragment deletions

The DELETE change pattern allows removing an existing fragment from a process model. This becomes challenging if the fragment contains interaction activities referring to other partners. If we do not update the processes of these partners when deleting the interaction activities, incompatibilities in the choreography are introduced. For example, a partner might then wait for a message that will never arrive or send a message that will never be consumed. To avoid such errors, a propagation mechanism should be adopted that keeps the processes (i.e., the public models of the partners) compatible with each other. Further note that the deletion of an interaction might have transitive (i.e. indirect) effects that cannot be solely handled based on the process structure; i.e., knowledge about semantics is required.

Example 7 We consider a supply chain scenario. Assume that a local city council starts a new construction project and hence collaborates with a city planner being in charge of the project execution. In turn, the city planner interacts with several third party partners responsible for designing, supplying and building tasks. Therefore, if the city council cancels the project, the city planner must cancel his contracts with the other partners as well.

**Fig. 8:**
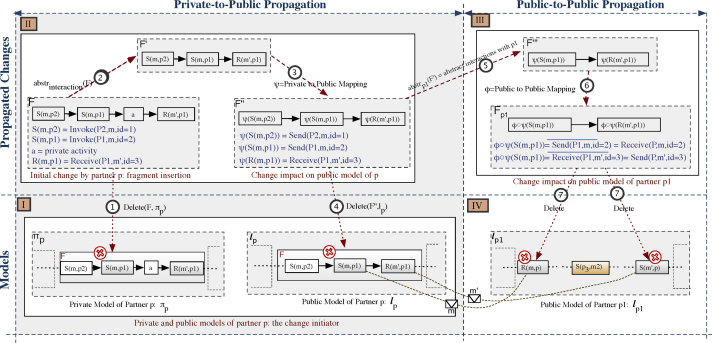
Major steps of propagating a fragment deletion.

This section does not consider the transitive effects of an interaction activity deletion, but only its direct structural effects. A non-exhaustive list of transitive scenarios as well as corresponding solutions are presented in Section 5. In the following, we use the example from Fig. 8 to illustrate the most important steps for propagating activity deletions. Given a partner process *π*_*p*_ and the fragment $F\in \pi_{p}$ to be deleted, we proceed as follows:

1. *Isolated or propagating changes*: We check whether F solely consists of private activities. In this case, the change can be considered as isolated and there is no need for any change propagation. If fragment F contains interaction activities, in turn, change propagation becomes necessary.
2. *Private-to-Public effects*: To determine the impact the deletion of the interaction activity has on the public model of the change initiator, we apply the following steps:
  • We identify all interaction activities to be deleted by abstracting F with respect to interactions; i.e. $F\prime ={\mathrm{abstr}}_{\mathrm{interaction}}(F)$ (cf. Step 2 in Fig. 8).
  • We identify the corresponding elements of F′ in the respective public model *l*_*p*_ of *p*. To this end, we use private-to-public mapping function *ψ* and delete all elements of F″=ψ(F) in *l*_*p*_ (cf. Steps 3–4 in Fig. 8).
3. *Public-to-Public effects*: To determine the change effects on the public models of the other partners, the following is applied: For each partner *p*_1_ involved in F″, we identify all interactions this partner is involved in by applying abstraction function $F\prime \prime \prime ={\mathrm{abstr}}_{p_{1}}(F\prime \prime )$. For each element of F‴, we determine the corresponding element in $l_{p_{1}}$ using the public-to-public mapping function *φ* (i.e., $F_{p_{1}}=\varphi (F\prime \prime \prime )$). Note that the elements of $F_{p_{1}}$ are not necessarily directly connected in $l_{p_{1}}$; they could be separated by other activities or gateways instead. To handle this case, for each of these elements we generate a separate delete operation. Finally, after each deletion, model refactorings may be applied (cf. Steps 5–7 in Fig. 8).

It is noteworthy that the interactions between two partners are often accomplished synchronously in the sense that the partner process who sends a message to another partner process may wait for a response from the latter before proceeding with its execution. In certain scenarios, it might happen that the response is deleted due to a transitive effect of the first deletion. We will discuss these transitivity issues in Section 5.

### 4.3 Propagation of fragment replacement

**Fig. 9:**
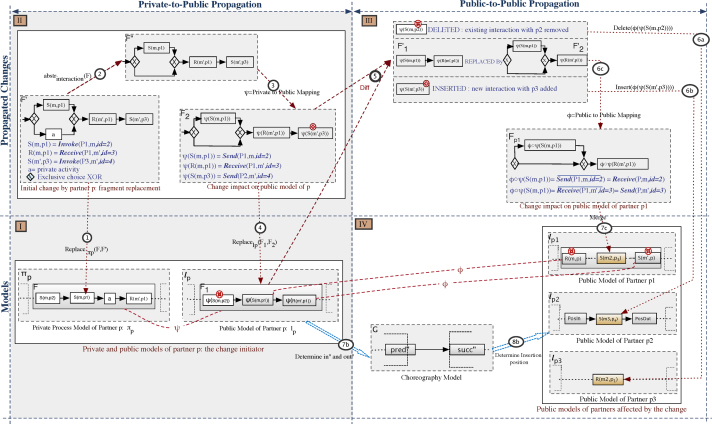
Major steps of propagating a fragment replacement.

Change pattern REPLACE modifies the structure and elements of a given fragment in a process model. This pattern is particularly useful when the redesign of the entire process or a part of it becomes necessary; e.g. to optimize the flow between the activities; e.g., in the *book trip* example from Fig. 1, one might want to replace fragment PAR(Airline_notification_failure, TravelAgency_notification_failure)by changing this parallel branching into a choice CHC(Airline_notification_failure, TravelAgency_notification_failure). In the following, we refer to the change scenario from Fig. 9 to illustrate the most relevant steps towards the propagation of the resulting changes. Given a fragment $F\in \pi_{p}$ to be replaced by a new fragment F′, change propagation can be accomplished as follows:

1. *Isolated or propagating changes*: We first need to determine whether the fragment replacement constitutes a local change or needs to be propagated to other partners as well. For this purpose, we check whether F or F′ contain any interaction activities. In this case, a propagation becomes necessary to maintain the compatibility between the partner processes. When replacing a fragment by another one, new interaction activities may be added, existing ones be removed, or the sequencing between interaction activities be changed. Accordingly, the partners directly affected by the fragment replacement are those interacting with *p* in the scope of both F and F′ (i.e., *p*_1_, *p*_2_ and *p*_3_ in the scenario from Fig. 9).
2. *Private-to-Public effects*: To identify the effects of a fragment replacement on the public model *l*_*p*_ of *p*, we first abstract fragments F and F′ with respect to interaction activities. Then, we identify the corresponding elements to be replaced in *l*_*p*_ using the private-to-public mapping function *ψ*; i.e. $F_{1}=\psi ○{\mathrm{abstr}}_{\mathrm{interaction}}(F)$ and $F_{2}=\psi ○{\mathrm{abstr}}_{\mathrm{interaction}}(F\prime )$. The initial replace request is then transformed into a ${\mathrm{REPLACE}}_{l_{p}}(F_{1},F_{2})$ operation that, in turn, needs to be propagated since it affects the interactions with other partners (cf. Steps 2–4 in Fig. 9).
3. *Public-to-Public effects*: To determine the change effects on the public models of the other partners, we do the following:
  • When replacing $F_{1}$ by $F_{2}$ (cf. Step 5 in Fig. 9), three scenarios are possible: (i) a partner involved in the original fragment $F_{1}$ is no longer present in the new fragment $F_{2}$ (i.e., the interaction with this partner is deleted), (ii) a partner involved in $F_{2}$ was not present in $F_{1}$ (i.e., a new interaction activity is added), and (iii) a partner is present in both fragments $F_{1}$ and $F_{2}$, but with different structure. Note that for one and the same replacement, we may have to deal with various scenarios of which each is related to a particular partner. Accordingly, we abstract both the new and the old fragments $F_{1}$ and $F_{2}$ with respect to each partner involved in the change. Accordingly, the REPLACE pattern is translated into a concatenation Δ of change patterns to be propagated to the concerned partners.
    (i) *Deletion scenario*: If a partner p′ interacts with partner *p* in the context of the original fragment $F_{1}$ and is not engaged in any interaction with *p* in the new fragment $F_{2}$, we delete the respective interaction activities from the public model of p′ (cf. Step 6a in Fig. 9). The deletion scenario is handled similarly as described in Section 4.2. (ii) *Insertion scenario*: If a particular partner p′ has no interactions with *p* in the context of old fragment $F_{1}$, but interacts with *p* in the new fragment $F_{2}$, we must insert the new interactions in the public model of p′ (cf. Steps 6b–8b in Fig. 9). The insertion scenario is propagated similarly as described in Section 4.1.
    (iii) *Replacement scenario*: The last scenario we consider is as follows: both fragments $F_{1}$ and $F_{2}$ involve interactions with partner p′, but with different structure. The latter means that the control flow dependencies between the interactions have changed and, therefore, the public model of p′ shall be updated to preserve compatibility between all public models (cf. Steps 6c–7c in Fig. 9). For example, in the change scenario from Fig. 9 and in comparison with $F_{1}$, $F_{2}$ keeps the same interaction activities with partner *p*_1_ for sending and receiving the messages *m* and m′, but with different structure; i.e., message *m* is not always sent due to the exclusive choice. Consequently, the public model of *p*_1_ should be updated and, in turn, the private model of *p*_1_ be adapted to the latter if needed. Formally, given $F_{1}$ and $F_{2}$, we apply an abstraction with respect to each partner involved in the change and compare both results. If ${\mathrm{abstr}}_{p\prime }(F_{1})={\mathrm{abstr}}_{p\prime }(F_{2})$ holds, no propagation to p′ is needed since the interactions with p′ remain invariant. Otherwise, a propagation is needed and the current interactions in $l_{p\prime }$ must be changed to ensure compatibility with the new fragment $F_{2}$.
    When propagating the changes to p′, first of all, we need to fetch the matching elements of $F_{1}$ in $l_{p\prime }$. In general, the interactions between *p* and p′ in the scope of the old fragment $F_{1}\in l_{p}$ do not always have the same structure or distribution in $l_{p\prime }$ (but the same behavior instead). This is due to the applied refactorings as well as the different interactions p′ has with the other partners; i.e., two interaction activities, which are directly connected in sequence in *l*_*p*_, are not necessarily directly connected in sequence in $l_{p\prime }$, but could be separated by an interaction activity not involving *p* instead. The same holds for an interaction activity surrounded by a parallel branch (i.e., AND) in $l_{p\prime }$, which could be refactored to a sequence in *l*_*p*_.
    Consider Fig. 9. If we look at $F_{1}^{\prime }$ and $F_{2}^{\prime }$ as the abstractions of $F_{1}$ and $F_{2}$ in respect to *p*_1_, matching activities of $F_{1}^{\prime }=\mathrm{Seq}(\psi (S(m,p_{1}))$, $\psi (R(m\prime ,p_{1})))\in l_{p}$ are R(m,p) and $S(m\prime ,p)\in l_{p_{1}}$. Note that these are separated by another interaction activity referring to *p*_2_. To integrate the change we must transform $F_{2}^{\prime }$, using the public-to-public mapping $F_{p_{1}}=\varphi (F_{2}^{\prime })$, and merge it with the smallest fragment containing R(m,p) and $S(m\prime ,p)\in l_{p_{1}}$ (i.e., the gray box in $l_{p_{1}}$ in Fig. 9).
    In general, we must consider the smallest fragment containing all interaction activities of $F_{1}^{\prime }$ in $l_{p}^{\prime }$. Then, we must merge it with the corresponding elements of $F_{2}^{\prime }$. For this, we must adopt an algorithm that merges two process models or fragments. Note that merging process models has been widely studied in literature [22,23]. The key idea is to merge different (and overlapping) process models into a single model without restricting the behavior represented in the original models. Formally, if we consider *γ*as a merge function, the problem can be solved by merging $F_{p_{1}}=\varphi (F_{2}^{\prime })$ with $\alpha_{l_{p_{1}}}(\varphi (F_{1}^{\prime }))$. It is noteworthy that such a merge might result in different scenarios among which the corresponding partner should chose the most appropriate one.

### 4.4 Further steps and discussion

This section discusses the change propagation approach and highlights the main steps that follow the public-to-public change propagation. Note that the following steps are outside the focus of this paper, but can be considered as complementary to our work.

*Negotiation*: Computing change effects on the public models of the partners is automatic, relying on the presented algorithms. As shown in Fig. 6, after this step, a negotiation phase is required to approve or reject the intended changes. In general, such a negotiation cannot be fully automated, but requires an agreement among the partners. In particular, negotiations may involve human actors, e.g., through phone, e-mail, or meetings. Various approaches [60,59] exist that have dealt with negotiations in the context of process choreographies (e.g., based on service level agreements). Finally, note that negotiations might result in a redefinition of the initial change.

*Public-to-private propagation*: The propagation of a process change to the partners׳ public models might require adaptations of their private models as well. In general, these adaptations cannot be determined by the partner that initiated the change. Accordingly, once all partners involved in the change have agreed on the public changes, each of them must determine the required changes of its private model. In particular, the new private model must be consistent with the changed public model. Note that changes of the partners׳ private processes, in turn, might lead to new changes that need to be propagated to other partners (i.e., transitivity). Since a change initiator must not access the private process of other partners, the partners affected by the change themselves are responsible for adapting their private processes to the requested change. In turn, this might lead to cascading effects or even the multiple involvement of a partner during change propagation.

*Change implementation*: After all public and private changes are determined and agreed on, the soundness of the corresponding models is checked, the changes are implemented, and the public, private and choreography models are updated.

In [35], a multitude of composite change operations are described of which not all are considered in this paper. In general, most change operations can be realized using the basic DELETE and INSERT operations; e.g., REPLACE can be considered as a combination of a DELETE followed by an INSERT. However, change propagation complexity varies significantly. Worst case, for example, the complexity of directly propagating a REPLACE is equal to the one of a DELETE followed by an INSERT. Indeed, the REPLACE operation refers to a fragment instead of a single node. Accordingly, replacing a fragment by a new one not means that all nodes of the old fragment are changed. Taking the nodes that remain unchanged into account significantly improves the propagation process and reduces the number of operations to be propagated. Regarding the REPLACE algorithm (cf. Algorithm 2), the three possible scenarios (i.e., deletion, insertion and replacement) are solely generated for parts that have changed. By contrast, unchanged parts do not require any propagation. However, a DELETE followed by an INSERT will first delete those parts, which entails a propagation to concerned partners, and then re-insert the same parts (entailing another propagation).

## 5 Transitivity of change propagation

This section presents a non-exhaustive list of use cases demonstrating the transitivity effects of the DELETE change pattern and the solutions to cope with them. Note that this is a semantic issue that cannot be resolved based on the propagation algorithms presented so far, which solely focus on structural issues. As example consider a scenario with three partners *p*_1_, *p*_2_ and *p*_3_. Assume that *p*_1_ invokes *p*_2_ and *p*_2_ invokes *p*_3_. The latter returns the intermediary result to *p*_2_, which then applies data transformations before sending the final result to *p*_1_. If now *p*_1_ decides to delete its interaction with *p*_2_, one must further delete the subsequent interaction between *p*_2_ and *p*_3_, which is solely used to deliver the final result. If a partner deletes an interaction, semantically, this means he is unable to afford this service anymore or he does not need the data anymore. Then, the challenge is to determine whether an interaction has transitive effects on other interactions, and if yes, to identify and resolve these transitive effects.

*Case* 1. Partner *p* is the *final consumer* of a data element, and it launches an interaction that requires a response. Accordingly, *p* contains related interaction activities *send* and *receive*. Thereby, *send* is used to request the data from another partner, whereas the corresponding *receive* is used to receive the response to this request from another partner.

**Fig. 10:**
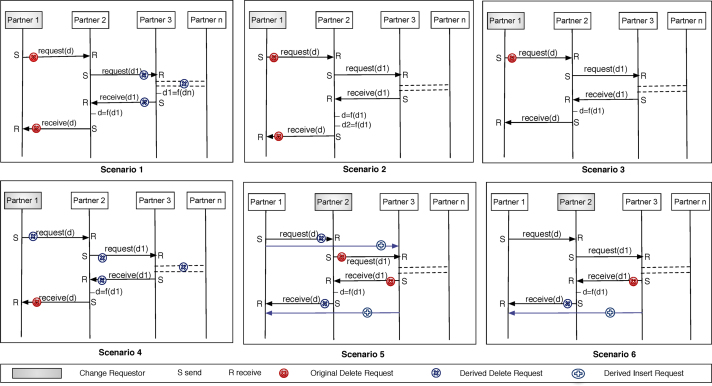
Transitivity scenarios.

• *Case 1.1 p* deletes the *send*/*receive* interaction activities; i.e., it does not need the data anymore (since *p* is the final consumer). Accordingly, we delete $\overset{¯}{\mathrm{send}}$ and $\overset{¯}{\mathrm{receive}}$. In case all subsequent interactions with other partners are *solely* used to deliver this data, these interactions are deleted as well (e.g. supply chain scenarios). Of course, it is also possible that only a subset of the subsequent interactions are used to deliver this data. These interactions are then deleted only if they do not have any other role in the choreography; i.e., they are not required to calculate any other data (cf. Scenario 1 in Fig. 10). If they play another role in the choreography, in turn, the subsequent interactions are kept (cf. Scenario 2 in Fig. 10).

Example 8 Assume that there are two concurrent requests from partners *A* and *B* to partner *C*. Further assume that *C* is involved in subsequent interactions and then replies to *A* and *B*. If *A* deletes its interaction with *C*, we must not delete the subsequent interactions of C since they are still required to reply to *B*.

• *Case 1.2 p* solely deletes the *send* pattern. We distinguish two scenarios:
  (i) Another partner starts the communication instead of *p*. Accordingly, we just update the corresponding $\overset{¯}{\mathrm{send}}$ with the new partner.
  (ii) *p* is not responsible anymore for triggering the other partner to deliver the response; i.e., the latter is provided automatically or under certain constraints. Hence, we delete the corresponding $\overset{¯}{\mathrm{send}}$ (cf. Scenario 3 in Fig. 10).
• *Case 1.3 p* solely deletes the *receive* pattern. This means either *p* does not need the data anymore or the latter is transferred to another partner. In the first case, we just delete the corresponding $\overset{¯}{\mathrm{receive}}$ and look for other interactions correlated with this response (used *solely* for delivering the response, cf. Scenario 4 in Fig. 10). In the second case, we update the corresponding $\overset{¯}{\mathrm{receive}}$ with the new partner.

*Case 2*. Partner *p* corresponds to the *final consumer* of the data, but is not responsible for launching the first interaction; i.e., *p* has only the *receive*. If *p* deletes the *receive* (i.e., *p* does not need the data anymore), we delete the corresponding $\overset{¯}{\mathrm{receive}}$ as well as all subsequent interactions that are *solely* used to deliver this data. All interactions participating in the delivery of this data, but having another role in the choreography, are kept.

*Case 3*. Partner *p* is the *starting point*, responsible for starting an interaction that results in a response to another partner; i.e., *p* has the *send*. Either (i) another partner is responsible for starting this interaction; then, we update the $\overset{¯}{\mathrm{send}}$ with the new partner, or (ii) the interaction starts automatically or under other constraints on the target partner; then we delete the corresponding $\overset{¯}{\mathrm{send}}$. Subsequent interactions are not deleted since we still need the final data to be delivered to the final consumer.

*Case 4*. Partner *p* is an *intermediary partner*, and has correlated interaction activities *receive* and *send. p* receives a request and starts a *subsequent* interaction necessary for delivering the final response to the requester.

• *Case 4.1* Partner *p* deletes both the *send* and the *receive* interaction activities and is unable to provide the data anymore. However, still the final response to the requestor is needed. In this case, two choices exist: (i) Looking for another partner that can take over the task of *p* to deliver this response. Then, we update the subsequent interactions as well as the ones of the root partner (i.e., the partner that invoked *p* and the one to whom *p* shall send the result) with this new partner. (ii) Deleting $\overset{¯}{\mathrm{send}}$ and $\overset{¯}{\mathrm{receive}}$ as well as all subsequent interactions *solely* used in the context of this *intermediary data*, and looking for another partner or set of partners that can provide this data. Then, we update the interactions with the root partners or *p*. As example consider Scenario 5 in Fig. 10. If *Partner*3 is able to accomplish the data transformation ($d=f(d_{1})$) of *Partner*2, the interactions of *Partner*1 with *Partner*2, which serve to deliver data *d*, are replaced by new ones with *Partner*3.
• Case 4.2 If *p* solely deletes the *send* interaction activity, another partner is responsible for starting this intermediary interaction or the subsequent interactions start automatically or under other constraints. In the first case, we update the corresponding $\overset{¯}{\mathrm{receive}}$ with the new partner, otherwise we just delete it.
• Case 4.3 If *p* solely deletes the *receive* pattern, this means that *p* cannot take over the tasks necessary to deliver the final data. (i) If other operations are necessary to deliver the final data, we look for another partner that can accomplish the same tasks. (ii) If not, we update the $\overset{¯}{\mathrm{Send}}$ to link it directly with the root partner (cf. Scenario 6 in Fig. 10).

*Conclusion*. We presented a non-exhaustive list of possible scenarios of transitivity when dealing with change propagation. Clearly, transitivity is a semantic issue and requires a data model defining the relationships between the exchanged data objects (e.g., an ontology). Due to privacy issues, in addition, not all data correlations are always known, and therefore calculating the transitivity effects remains problematic and cannot be fully automated. Several proposals exist to predict the transitive effects in process choreographies based on prediction metrics (e.g. social graphs) [10].

## 6 Compatibility and consistency

This section discusses soundness issues of a process choreography in the context of change propagation. In particular, we check whether the compatibility and consistency properties of the collaborating business partners are kept. Accordingly, we assume that the initial public models of the collaborative processes are compatible with each other and that each private model is consistent with its corresponding public model. We further assume well-behavedness of the change operation in terms of structure and semantics. Recently, several proposals were made on checking the soundness of choreographies in terms of compatibility and consistency [15,24–28].

Before discussing the compatibility and consistency of the process choreography in the context of change propagation, first of all, we introduce useful properties. Thereby, Property 1 states that for each node of the public model of a partner *p*, there should be a matching element in the corresponding private model of *p*, but not vice versa. Furthermore, Property 2 expresses that for each node of the public model of a partner *p*, there should be a matching node in a different public model of another partner. Note that this is a necessary, but not yet sufficient condition for ensuring compatibility between public models. Finally, Property 3 states that for each node in a public model, there should be a matching node in the choreography model. In particular, for each interaction in the choreography model, there should be exactly two matching interaction activities in the public models. Formally:

Property1 $\forall l\_\mathrm{node}\in l_{p},\exists p\_\mathrm{node}\in \pi_{p}\;\mathrm{with}\;\psi (l\_\mathrm{node})=p\_\mathrm{node}$.

Property 2 $\forall l\_\mathrm{node}\in l_{p}\;\mathrm{with}\;\mathrm{type}$ (*l*_*node*)=*InteractionActivity*: $\exists p\prime \neq p:\exists l\_\mathrm{node}\prime \in l_{p\prime }$ *with type* (l_node′)=InteractionActivity∧φ(l_node)=l_node′.

Property 3 $\forall l\_\mathrm{node}\in l_{p}:\exists c\_\mathrm{node}\in G$ *such that* ξ(l_node)=c_node.

Lemma 1 ${\mathrm{abstr}}_{p\prime }(F)\in L_{p}⟹\overset{¯}{{\mathrm{abstr}}_{p\prime }(F)}\in {\mathrm{abstr}}_{p}(L_{p\prime })$. *The complement of the abstraction of a fragment* $F\in L_{p}$ *from the perspective of a participant* p′ *is a fragment of the abstraction of* $L_{p\prime }$ *according to p*.

Proof The proof of this lemma can be based on the following compatibility properties of choreographies. (c.f. [15]).

• If $a\in L_{p}$ corresponds to an activity that interacts with partner p′, the following holds: $\exists b\in L_{p\prime }$ with $b=\overset{¯}{a}$.
• If *a*_*i*_, $a_{j}\in L_{p}$ are two activities interacting with the same partner p′ and $\beta (a_{i},a_{j})$ is a function returning the minimal precedence relation (i.e., control flow path) between *a*_*i*_ and *a*_*j*_ [21], the following property (also denoted as bi-simulation property [15,18]) holds:
  $$\exists b_{i},b_{j}\in L_{p\prime }\;\mathrm{with}\;b_{i}=\overset{¯}{a_{i}},\;b_{j}=\overset{¯}{a_{j}}\wedge \beta (a_{i},a_{j})=\beta (b_{i},b_{j})\;□$$

### 6.1 Consistency checking

In our context, consistency means that the implementation of a business process (i.e., a private model) is consistent with its observable behavior (i.e., public model). This ensures that implementations of private processes satisfy the interaction constraints defined in the public models [15]. In our change propagation approach, the public model is defined as an abstraction of the private model by deleting all model elements not related to any interaction (e.g., Property 1). Accordingly, an insertion, deletion or replacement of a fragment in a private model needs to be transformed into an insertion, deletion or replacement of the fragment abstraction in the public model (if required). Since any abstraction preserves the consistency between the original and abstracted model (cf. [29,30]), the propagation from private-to-public does not affect consistency. Regarding deletion or replacement scenarios, refactorings may be applied. In turn, this eliminates unnecessary synchronization elements (e.g. a parallel branching between an activity and an empty branch is reduced to a sequence), but does not affect the consistency between the original and abstracted model. Change propagation might also result in the insertion, replacement or deletion of a fragment from a public model of a partner target. If the change is accepted by the latter, the change requester cannot check for the consistency between the public and private model of that partner since the private model of the latter is not visible. Our approach assumes that any partner affected by the change should update his private model locally if he accepts the change request. In turn, this update must be consistent with the new version of his public model.

### 6.2 Compatibility checking

Compatibility is a soundness criteria that checks whether the interacting partners are able to communicate with each other in a proper way (e.g., no deadlocks or livelocks will occur). In this context, [15] distinguishes between structural and behavioral compatibility:

*Structural compatibility*: It requires that for every message that may be sent, the corresponding partner is able to receive it. In turn, for every message that can be received, the corresponding partner must be able to send a respective message. Regarding our propagation mechanism, structural compatibility is always preserved. Depending on the change operation type, for each affected partner we add, update, or remove the complement of what has been changed in the process of the change initiator. In particular, for each interaction activity *send* in one process partner source, we insert or delete the corresponding *receive* interaction activity with the expected attributes (e.g. message) in the process of the partner target (i.e., affected by the change) and vice versa (cf. Properties 2 and 3, and Lemma 1).

*Behavioral compatibility*. It considers behavioral dependencies (i.e., control flow) between message exchanges, i.e, it deals with the ordering of the partners׳ interactions. For example, a *Receive* encapsulated by a *Sequence* in one partner process should not be linked to a *Send* encapsulated by a *Choice* in the process of a different partner. Indeed, this might lead to a deadlock in case the path containing the *Send* in the *Choice* is not executed during runtime.

Assume that $\left(\delta ,\pi_{p}\right)$ is the change operation to be applied to process model *π*_*p*_ and $\left(\delta ,L_{p}\right)$ corresponds to the inferred change to be applied to the public model of *p*. Further, let Δ be the set of changes inferred from $(\delta ,L_{p}$) to be propagated to its directly affected partners. For each affected partner $p_{i}$, $\left(\delta_{i},L_{i}\right)$ represents the inferred change operation to be propagated to its public model; i.e., $\Delta =\wedge_{i=1‥n}(\delta_{i},L_{i})$, where *n* corresponds to the number of affected partners. Note that the number of inferred changes is finite since we only consider propagations to direct partners. In turn, changes that might have structural effects on other partners (due to transitive relations) are propagated to them through their direct partners recursively.

If $\left(\delta ,L_{p}\right)$ is invariant (i.e., it does not affect the public model of *p*), consistency and compatibility are preserved over the collaborative partners. In addition, since both processes and changed fragments are structured, consistency and compatibility relations can be reduced to those existing between the fragments affected by the change.

• The INSERT pattern augments the process models of the partners affected by the change with new activities and gateways respectively. Further, it does not affect the structural or behavioral dependencies (i.e., control flow) between the existing activities. However, some direct precedence relations between activities may be transformed into transitive ones (due to the insertion of new activities and gateways). The propagation of a change operation of type INSERT results solely in change operations of type INSERT in the public models of the affected partners. According to a particular partner, the insertion is done with respect to the direct and transitive dependencies with the activities of the same partner. As explained in Section 4, if F corresponds to the fragment to be inserted in $L_{p}$, $\overset{¯}{{\mathrm{abstr}}_{i}(F)}$ is the fragment to be inserted in $L_{i}$. Note that the latter shows the same behavior (i.e., control flow) as ${\mathrm{abstr}}_{i}(F)$. In turn, the insertion position is computed based on the transitive *preset* and *postset* of $F_{i}$ with respect to partner i. Note that this preserves the order of the fragments and ensures their behavioral compatibility after propagating the INSERT operation. This propagation might result in a merge of the fragment to be inserted with an existing fragment as described in Algorithm 3. In particular, if the partner affected by the change interacts with different partners in the scope of the calculated insertion positions (i.e., there exist others interactions activities between the identified positions *pred* and *succ*), the fragment to be inserted between these two positions must be merged with the existing interaction activities in between. Accordingly, we assume that merge function gamma:(F,F′)→F″ preserves the behavior of F and F′ in the result F″ of the merge.
• The DELETE operation reduces the process models of the partners affected by the change. This reduction is accomplished in a symmetric way on both sides; i.e., *p* and the partners affected. The deletion of an activity on one side results in the deletion of the corresponding $\overset{¯}{a}$ on the other. Structural and behavioral compatibilities are kept. However, other issues emerge, e.g., an activity might wait for data that will never arrive or send a message that might not be consumed. The solution we proposed in Section 5 deals with typical use cases where the correlated interactions are updated or deleted accordingly. Note that this neither affects structural nor behavioral compatibility of the propagation.
• The propagation of the REPLACE operation results in three scenarios: insert, delete, or merge. Assume that the merge function *γ* is correct and idempotent, preserving the behavior of the merged fragments. We consider $F_{i}$ and $F_{i}^{\prime }$ as the fragments to be merged. Then, the behavior of $F_{i}$ is reflected by the merge result $\gamma (F_{i},F_{i}^{\prime })$ (cf. Lemma 1).

The consistency between the public and private models of the partners affected by the change will be checked if negotiations succeed. Each of these partners must then adapt its private model to the change of its public model. Using consistency rules, each partner can check locally whether or not its private model is consistent with its public model. More details about consistency checking and the validation of choreographies can be found in [5,2].

## 7 Proof-of-concept prototype and validation

This section outlines the architecture of our change propagation framework for process choreographies and presents the prototypical implementation of the *C*^3^Pro Editor as one of its core components.

### 7.1 Framework architecture

**Fig. 11:**
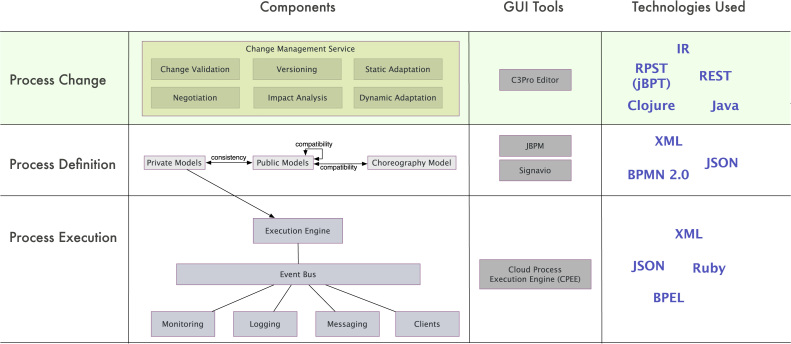
Architecture of the change propagation framework.

Our architecture must provide functions for defining and executing process choreographies. Further, it must allow specifying, performing and propagating changes. To meet these requirements, we propose a layered architecture as depicted in Fig. 11. It consists of three layers: *Process Definition*, *Process Change*, and *Process Execution*.

The main change propagation functions are realized by the *Process Change* layer. The $C^{3}\mathrm{Pro}$ Editor is one of the core components of this layer that realizes the change propagation algorithms presented in Section 4. Other functionalities provided by existing tools are delegated (e.g. process modeling and execution); i.e., although we focus on the functionalities of the *Process Change* layer, we communicate with the other two layers as well.

In the *Process Definition* layer, process designers use existing modeling tools (e.g., Signavio or jBPM) to create process as well as choreography models. The latter are serialized as XML or JSON files and serve as input for the *Process Change* layer. In turn, the latter layer defines all components related to change propagation in choreographies. Most prominently, the Change Management Service implements Algorithms 3–2 as well as the internal representation (IR) of private, public and choreography models. The functions of the other components from the *Process Change* layer are follows:

• Versioning capabilities are provided that allow *undoing* as well as *redoing* changes.
• Dynamic adaptation is enabled to deal with the migration of running process instances.
• Model verification is supported to verify the soundness of the models resulting after a change.
• Negotiation becomes necessary if a change is not acceptable for a partner. This component deals with strategies applicable if a negotiation is required (cf. Fig. 6).

All functions provided by the *Process Change* layer are exposed as a RESTful service, which allows for a unified access from any client able to communicate via HTTP. The Change Management Service can be accessed with the $C^{3}\mathrm{Pro}$ Editor serving as the connector to the *Process Definition* layer. The $C^{3}\mathrm{Pro}$ Editor provides functions for importing and visualizing choreography models. Moreover, changes may be applied to the models and required change propagations to partners be performed, allowing for the simulation of change propagation. Altogether, the $C^{3}\mathrm{Pro}$ Editor serves as the front end for all components defined in the *Process Change* layer.

The Change Management Service serves as a pluggable middleware based on which process engines can be integrated. In particular, this integration allows these engines to access all components of the *Process Change* layer. This implies that after a successful change propagation, which includes negotiation and soundness checks (cf. Fig. 6), the updated choreography models are transformed into an executable form being directly passed to the process engine for enactment (*Process Execution* layer). In other words, from the perspective of the *Process Change* layer, the *Process Execution* layer serves as an execution platform for the updated models. The process execution engine we have chosen is the Cloud Process Execution Engine (CPEE).

### 7.2 Tool support: $C^{3}\mathrm{Pro}$ editor

In the following, all occurrences of *nodes* refer to *PNode* (i.e., activities and control nodes) from Definition 3. We implemented the $C^{3}\mathrm{Pro}$ Editor as the first prototypical client realizing the Change Management Service. In particular, this client component takes the role of a simulation environment for manually stepping through the change propagation process (cf. Fig. 6), which allows testing and verifying change scenarios. Altogether, the $C^{3}\mathrm{Pro}$ Editor supports the visualization of

1. private, public and choreography models,
2. affected partners׳ *nodes* and fragments depending on the change type (i.e., INSERT, DELETE or REPLACE), and
3. the models resulting after the application of the calculated changes.

A multitude of process modeling tools exist. For this reason, we delegate the basic modeling functions to these tools. In our case, we have used Signavio [14]. We export the created models to BPMN 2.0 XML format. In turn, the latter is directly supported by our change propagation library for importing models. Once imported, models can be visualized and all changes be performed with the $C^{3}\mathrm{Pro}$ Editor. The latter is accomplished by propagating the changes to each affected partners. The $C^{3}\mathrm{Pro}$ Editor not only visualizes process models before and after a change, it also displays auxiliary information such as the affected partners׳ nodes and fragments. We utilize the jBPT^4^ library for handling the transformation of models (private, public and choreography) to RPST.

We realized the Change Management Service as a Java Library (JAR), which enables any language running on top of the Java Virtual Machine (JVM) to access the underlying public classes and static functions. This allowed us to develop the $C^{3}\mathrm{Pro}$ Editor in a rapid fashion, still treating the Change Management library as a service. A frequently changing API would have hindered the concurrent development of the service and the editor. After finalizing the API and identifying the required functionalities, we implemented the REST infrastructure for the Change Management Service the $C^{3}\mathrm{Pro}$ Editor consumes.

We chose Clojure as programming language that is amendable for rapid prototyping and iterative development. Further, it has a rich Read Eval Print Loop (REPL) environment. Its default runtime platform is JVM, enabling seamless interoperability with the Change Management service. Clojure follows a functional programming style and provides concurrent functionalities; the latter are important for GUI application development. Finally, it allows changing the behavior of a running program without restarting it and hence reducing development efforts significantly.

**Fig. 12:**
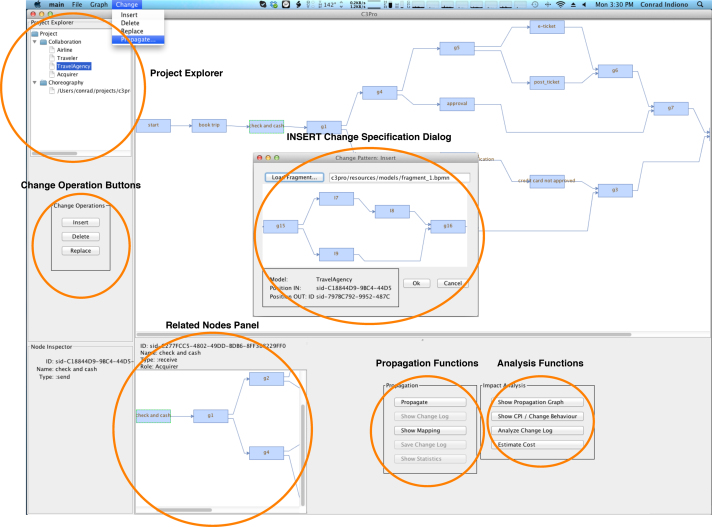
$C^{3}\mathrm{Pro}$ Editor—screenshot showing an INSERT operation being performed.

**Fig. 13:**
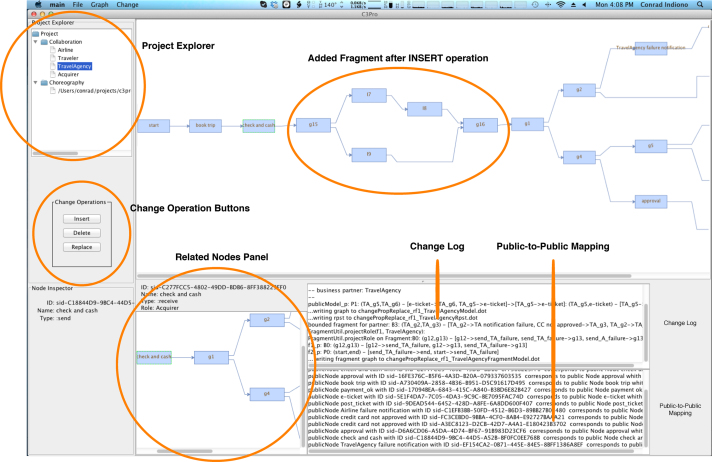
$C^{3}\mathrm{Pro}$ Editor—screenshot showing a public model after an INSERT operation.

Figs. 12 and 13 depict screenshots of the $C^{3}\mathrm{Pro}$ Editor. The *Project Explorer* on the left-hand side shows the current choreography model as well as the public models of all partners participating in the collaboration. Double clicking on any one of these items will display the corresponding model as a graph in the *Graph Panel*. In turn, the *Graph Panel* visualizes the selected graph as expected. Left-clicking on a *node* in the displayed graph will bring up its detailed information in the *Detail Panel* and show the related nodes in the *Related Nodes Panel*. The related *node* depends on the currently selected one. For each *send message*, the associated *receive message* is found and displayed, and vice versa. If an interaction activity (i.e., a *node* of the choreography model) is selected, the actual send and receive messages are picked from the appropriate public models and displayed in the *Related Nodes Panel*. If gateways (i.e., *ControlNode* of Definition 3) are selected, the smallest fragment (see Definition 9) that surrounds the selected gateway is displayed in the *Related Nodes Panel*.

Change operations are shown to the user by right-clicking on a node within the *Graph Panel* as well as on the left side below the *Project Explorer* (as buttons). When clicking one of the provided operations, a dialog window pops up prompting the user to specify the change. In the scenario depicted in Fig. 12, the user is asked to load the fragment for the INSERT operation. Afterwards, the $C^{3}\mathrm{Pro}$ Editor applies the change and triggers the change propagation process required. Fig. 13 shows the screen after applying an INSERT operation. Furthermore, the *Graph Panel* allows for the display of a process model in terms of an RPST. Finally, the *Change Log* shows the output during the processing of a change propagation.

### 7.3 Implementation of the trip booking process

As we were unable to find publically available choreography models that can serve as the basis for our simulation, we opted to use the trip booking example (cf. Figs. 1–3). We used the Signavio Process Editor to model both the choreography models and the partner-specific public models of the choreography. Note that it was ensured that all models are structurally as well as behaviorally sound. Further on, we ensured that the process models are block-structured, which, in turn, allowed for their easy transformation into corresponding RPST representations. In case unstructured models shall be imported, the techniques described in [18] can be applied to transform most of these models into structured ones.

Models are exported as XML files and then imported as initial data set into the $C^{3}\mathrm{Pro}$ Editor. In total, 17,068 change operations of type INSERT, DELETE, and REPLACE were created and tested on the prototype.

## 8 Related work

Change propagation has been an active research area in software engineering. In particular, the analysis, evaluation and propagation of changes have been widely studied in large complex software systems [31–34,36–38]. However, respective approaches cannot be directly transferred to process choreographies since the latter entail several particularities; e.g., the distributed model structure, partly unavailable information about partner processes, dynamic aspects, and specific requirements (e.g., compliance, privacy and security). Note that these particularities raise additional challenges for change propagation algorithms, which have been partially addressed by only few approaches so far.

In [39], four transfer rules for dealing with dynamic changes of distributed processes are proposed. These rules use projection/protocol and life cycle inheritance relations in order to check whether a changed process corresponds to a subclass of the original one. The suggested method solely allows for changes preserving inheritance transformation rules, i.e., changes having only internal effects. Particularly, there is neither a need for change propagation nor for any new agreements on the global protocols (i.e., the choreography model) since only inheritance-conforming changes are allowed. By contrast, our approach also supports changes that affect the external behavior of a process by computing and propagating them to the affected partners.

In [40,41], change propagation techniques for partitioned processes are proposed, where a process model is split into several distributed partitions. This approach propagates changes applied to the original model to the respective partitions. It uses a decentralization function to compute the affected partitions and to infer the changes to be propagated as well. Moreover, it is one organization controlling the original as well as the derived partitions. Hence, it becomes easier to exactly compute the affected regions and the changes to be applied. Note that this differs from our approach since changes are applied by one partner participating in the choreography and are then propagated to the others. In particular, our work considers fully distributed processes; i.e., no partner holds information about another partner׳s private model. Each partner can only view the public models of the other partners. In the context of changes, this requires a negotiation phase between the affected partners and could have transitive structural and semantical effects on other partners recursively. As opposed to [40,41], our work considers transitivity issues as well. Other approaches similar to [40] are presented in [42,43].

The DYCHOR framework [9] addresses the challenge of propagating changes in process choreographies as well. Thereby, changes are classified into additive and subtractive changes, which may have variant or invariant impact on the interactions. DYCHOR uses annotated finite state automata to model choreographies and employs a set of operators to compute the changes to be propagated. In our work, we propose four change patterns that deal with more complex fragments instead of single activities solely. This leads to particular challenges concerning semantical and structural transitivity effects, and also requires negotiations with the partners affected by the change. We sketched semantic transitive effects as well as the solutions to deal with them. The approach adopted in this paper makes change propagation easier since process models are structured and only changed regions are affected. Hence, there is no need for completely re-computing the public models of affected partners entirely. Instead only the affected regions need to be adapted.

In [44], the problem of dynamic changes and versioning of process models is addressed. The same challenge is tackled in [45], where an ontology-based framework for decentralized workflow change management is presented and different migration rules for dynamic change adaptation are defined. In [46], change propagation between semantically overlapping process models, whose elementary as well as complex correspondences have been identified, is proposed. All these approaches are complementary to our work.

In [47], a method for propagating changes applied to a given software model is presented. In particular, it computes the additional changes required to meet an emerging change requirement. The approach proposes techniques to deal with consistency constraints violation. Different repair solutions (i.e., customizations) are introduced using cost models. In this approach, UML (Unified Modeling Language) is employed to specify the software model; further OCL (Object Constraint Language) is used to define constraints. [48] represents an extension of [47] to support SOA (Service Oriented Architecture); it is shown how changes can be propagated across a number of models using the Service-oriented Modeling language (SoaML). The cost calculation is substituted by a minimal modification strategy that helps selecting change options in such a way that it accommodates both the structural and semantic dimensions of SOA models.

Mafazi et al. [49] present an approach to propagate changes between *process views*. This approach considers a *reference model* from which several process views are derived. Further, it uses Petri nets to represent the different models, as well as means to check consistency after change propagation. A similar approach is provided in [50]. The models adopted by these approaches are different from the one described in this paper, where we distinguish between the private, public and choreography models. Accordingly, we distinguish between the compatibility public-to-public and the consistency private-to-public. Further, [49] does not consider the transitive effects of propagation. A similar approach is presented in [46], which computes change propagation between process views. However, the relationships between the activities of the different process views and the corresponding reference model are not explicit. This approach uses behavioral profiles to identify changed regions.

Mahfouz et al. [51] present an approach towards the customization of interactions in choreographies. It adopts TROPOS [52] to represent organizational business requirements. A new business requirement of a partner leads to a customization of the choreography model, which, in turn, results in a customization of the public models of these partners. Though [51] describes the general conceptual approach of propagation, it is unclear how the affected regions and changes to be propagated are determined. Finally, neither the change patterns nor the transitivity effects to be handled are discussed in [51].

In [53], an approach for aligning and propagating changes between the business process model and the corresponding service-component configuration model (SCA) is presented. The purpose of this approach is different from ours since it does not consider the change propagation between different process models, but between the business logic and its supporting software architecture logic instead (i.e., its implementation).

Recently, approaches started to analyze propagation effects when applying changes in process choreographies. As after a propagation changes cannot be imposed on affected partners, but are often subject to negotiations, propagation failures might become expensive. First steps towards the understanding of the ripple effects of change propagation in choreographies are taken in [10,11], where [10] operates on the choreography model structure and [11] on change log information by applying memetic mining.

Solutions to check the realizability of the choreography in case a specific reconfiguration or a change is needed are described in [54]. In particular, this allows avoiding changes that affect the realizability of the choreography. For this purpose, choreography models are translated into the FSP process algebra. In [55,56], an approach to model and validate *compliant choreographies* is presented, and techniques to check *compliance rules* in the context of process choreographies are defined. Note that these approaches, combined with change propagation algorithms, can be complementary to our work for ensuring sound propagations.

## 9 Summary and outlook

While business process management has reached a mature level in respect to enterprise-wide processes, the operational support of cross-organizational processes still constitutes a big challenge. In many application domains, however, any technology support will not be accepted if it is unable to cope with process changes and the evolutionary nature of business processes. This was confirmed in several case studies we conducted in the automotive domain (e.g., cross-organizational processes for product change management [61] and product release management [62]) as well as in the healthcare domain (e.g., cross-organizational processes coordinating the various healthcare partners involved in the preparation and enactment of a complex surgery [63]). Nevertheless, these case studies have also revealed the high need for a flexible support of cross-organizational processes.

This paper provides algorithms for propagating process changes in collaborative scenarios that involve multiple partners. In order to stay independent from a particular process specification language, RPST is used for defining public and private models of the involved partners as well as the choreography model. The proposed propagation algorithms consider typical process change patterns such as INSERT, DELETE, and REPLACE, and are evaluated based on their structural as well as behavioral compatibility.

Certain assumptions are made in this paper. First, the proposed algorithms consider the application of one change operation at a time. However, in practical scenarios, several change operations might be applied in a combined manner within a change transaction. To incorporate such complex changes, optimizations on the change transactions as suggested in [57] may be utilized. These allow calculating the actual effects of the change transaction. Second, change propagation might become necessary in a transitive way, i.e., multiple partners might be affected. This can be handled by applying the change propagation procedure depicted in Fig. 6 iteratively. However, it must be considered whether the transitive propagation becomes cyclic. In this case, mechanisms such as upper bounds on the number of iterations of propagating changes including rollback mechanisms are conceivable.

Currently, we are integrating the change propagation algorithms proposed into our cloud-based process execution engine CPEE. Further, we aim to test and apply these algorithms in future case studies with our partners from the automotive domain. As future work, we will deal with negotiation and public-to-private change propagation issues as well. Although our approach is able to determine the effects changes of a private model have on the public models of the involved partners, the dynamic effects on the running instances have not been considered yet. Therefore, as a next step, we aim to explore the effects of dynamic changes in the context of choreographies, as well as their impact on already running instances. Furthermore, the presented approach mainly deals with structural changes of choreographies and the resulting effects. However, it will be also interesting to extend the choreography models with data semantics (e.g., an ontology of the used data objects) to better cope with the transitive effects of changes. Finally, choreography version management as well as semantic constraints for choreography changes (i.e., to preserve global compliance rules in the context of changes [55]) will be investigated.

## Figures and Tables

**Table:** 

**1**	**Input**: - A Choreography C
**2**	- The fragment $F\in \pi_{p}$ to be deleted
**3**	**begin**
$\begin{array}{c} 4\\ 5\\ 6\\ 7\\ 8\\ 9\\ 10\\ 11\\ 12\\ 13\\ 14\\ 15\\ 16\\ 17 \end{array}$	$|\begin{array}{c} \mathrm{if}\;({\mathrm{abstr}}_{\left(\mathrm{interaction}\right)}(F)\neq \emptyset )\;\mathrm{then}\\ |\;\mathrm{update}\pi_{p}\;//\;\mathrm{local}\;\mathrm{change}–\;\mathrm{no}\;\mathrm{propagation}\;\mathrm{is}\;\mathrm{needed}\\ \mathrm{else}\\ |\begin{array}{c} \Delta \leftarrow \emptyset //\mathrm{decomposition}\;\mathrm{result}\\ P_{\Delta }\leftarrow \;\mathrm{List}\;\mathrm{of}\;\mathrm{all}\;\mathrm{business}\;\mathrm{partners}\;\mathrm{involved}\;\mathrm{in}\;F\\ F\prime \prime \leftarrow \psi ○{\mathrm{abstr}}_{\left(\mathrm{interaction}\right)}(F)\\ \mathrm{for}\;\mathrm{each}\;p\prime \in P_{\Delta }\;\mathrm{do}\\ |\begin{array}{c} F_{p\prime }\leftarrow {\mathrm{abstr}}_{p\prime }(F\prime \prime )\\ \mathrm{for}\;\mathrm{each}\;n\in F_{p\prime }\;\mathrm{do}\\ \left|\Delta \leftarrow \Delta \wedge (\mathrm{Delete}(\varphi (n)),l_{p\prime }\right)\\ \mathrm{end} \end{array}\\ \;\;\;\;\;\mathrm{end} \end{array}\\ \;\mathrm{end}\\ \;\mathrm{Output}:\Delta \end{array}$
**18**	**end**

**Table:** 

**1Input**: - Choreography C
**2** - The old fragment to be replaced $F\in \pi_{p}$
**3** - The new fragment F′
**4begin**
$\begin{array}{c} 5\\ 6\\ 7\\ 8\\ 9\\ 10\\ 11\\ 12\\ 13\\ 14\\ 15\\ 16\\ 17\\ 18\\ 19\\ 20\\ 21\\ 22\\ 23\\ 24\\ 25\\ 26\\ 27\\ 28\\ 29\\ 30\\ 31\\ 32\\ 33\\ 34\\ 35 \end{array}$$|\begin{array}{c} \Delta \leftarrow \emptyset //\mathrm{decomposition}\;\mathrm{result}\\ P_{\Delta }\leftarrow \;\mathrm{List}\;\mathrm{of}\;\mathrm{all}\;\mathrm{partners}\;\mathrm{involved}\;\mathrm{in}\;F\cup F\prime \\ \mathrm{if}\;(F=\emptyset \;\mathrm{and}\;F\prime =\emptyset )\;\mathrm{then}\\ |\;\mathrm{invariant}\;\mathrm{change}–\;\mathrm{no}\;\mathrm{propagation}\;\mathrm{needed}\\ \mathrm{else}\\ |\begin{array}{c} F_{1}\leftarrow \psi ○{\mathrm{abstr}}_{\left(\mathrm{interaction}\right)}(F)\\ F_{2}\leftarrow \psi ○{\mathrm{abstr}}_{\left(\mathrm{interaction}\right)}(F\prime )\\ \mathrm{for}\;\mathrm{each}\;(\mathrm{partner}\;p\prime \in P_{\Delta })\;\mathrm{do}\\ |\begin{array}{c} \mathrm{if}\;{\mathrm{abstr}}_{p\prime }(F_{1})\neq {\mathrm{abstr}}_{p\prime }(F_{2})\;\mathrm{then}\\ |\begin{array}{c} \mathrm{if}\;{\mathrm{abstr}}_{p\prime }(F)=\emptyset \;\mathrm{then}\\ |\begin{array}{c} /⁎\;\mathrm{Insertion}\;\mathrm{Scenario}:\;\mathrm{call}\;\mathrm{for}\;\mathrm{insert}\;\mathrm{propagation}\;\mathrm{algorithm}⁎/\\ \Delta \leftarrow \Delta \wedge \mathrm{Insert}\_{\mathrm{Propag}}_{\pi_{p}}({\mathrm{abstr}}_{p\prime }(F\prime ),\mathrm{Preset}(F),\mathrm{Postset}(F)) \end{array}\\ \mathrm{else}\\ |\begin{array}{c} \mathrm{if}\;{\mathrm{abstr}}_{p\prime }(F\prime )=\emptyset \;\mathrm{then}\\ |\begin{array}{c} /⁎\;\mathrm{Deletion}\;\mathrm{Scenario}:\;\mathrm{call}\;\mathrm{for}\;\mathrm{delete}\;\mathrm{propagation}\;\mathrm{algorithm}⁎/\\ \Delta \leftarrow \Delta \wedge \mathrm{Delete}\_{\mathrm{Propag}}_{\pi_{p}}({\mathrm{abstr}}_{p\prime }(F)) \end{array}\\ \mathrm{else}\\ |\begin{array}{c} /⁎\;\mathrm{Replacement}\;\mathrm{Scenario}⁎/\\ F_{1}^{\prime }\leftarrow {\mathrm{abstr}}_{p\prime }(F_{1})\\ F_{2}^{\prime }\leftarrow {\mathrm{abstr}}_{p\prime }(F_{2})\\ F_{p\prime }\leftarrow \gamma (\alpha_{l_{p\prime }}(\varphi (F_{1}^{\prime })),\varphi (F_{2}^{\prime }))/⁎\;\gamma \;\mathrm{is}\;a\;\mathrm{merge}\;\mathrm{function}⁎/\\ \Delta \leftarrow \Delta \wedge \mathrm{REPLACE}(F_{1}^{\prime },F_{p\prime },l_{p\prime })\\ \mathrm{for}\;\mathrm{each}\;(\mathrm{activity}\;a\in F_{1}^{\prime }\;\mathrm{such}\;\mathrm{that}\;a\notin F\prime )\;\mathrm{do}\\ |\Delta \leftarrow \Delta \wedge {\mathrm{DELETE}}_{p}(a)/⁎\;\mathrm{model}\;\mathrm{refactoring}\;\mathrm{may}\;\mathrm{be}\;\mathrm{applied}⁎/\\ \mathrm{end} \end{array}\\ \mathrm{end} \end{array}\\ \mathrm{end} \end{array}\\ \mathrm{end} \end{array}\\ \mathrm{end} \end{array}\\ \mathrm{end}\\ \mathrm{Output}\;\Delta ; \end{array}$
**36end**

**Table:** 

**1Input**: - A Choreography C (cf. Definition 4)
**2** - The fragment F to be inserted in *π*_*p*_
**3** - The insertion position *pred* and $\mathrm{succ}\in \pi_{p}$
**4begin**
$\begin{array}{c} 5\\ 6\\ 7\\ 8\\ 9\\ 10\\ 11\\ 12\\ 13\\ 14\\ 15\\ 16\\ 17\\ 18\\ 19\\ 20\\ 21\\ 22\\ 23\\ 24\\ 25\\ 26\\ 27\\ 28\\ 29\\ 30\\ 31\\ 32\\ 33\\ 34\\ 35\\ 36\\ 37\\ 38\\ 39\\ 40\\ 41\\ 42\\ 43\\ 44\\ 45\\ 46\\ 47\\ 48 \end{array}$$|\begin{array}{c} \mathrm{if}\;({\mathrm{abstr}}_{\left(\mathrm{interaction}\right)}(F)\neq \emptyset )\;\mathrm{then}\\ |\mathrm{update}\;\pi_{p}//\mathrm{local}\;\mathrm{change}–\mathrm{no}\;\mathrm{propagation}\;\mathrm{is}\;\mathrm{needed}\\ \mathrm{else}\\ |\begin{array}{c} \Delta \leftarrow \emptyset //\mathrm{The}\;\mathrm{set}\;\mathrm{of}\;\mathrm{changes}\;\mathrm{to}\;\mathrm{be}\;\mathrm{propagated}\\ P_{\Delta }\leftarrow \;\{\mathrm{Set}\;\mathrm{of}\;\mathrm{all}\;\mathrm{business}\;\mathrm{partners}\;\mathrm{involved}\;\mathrm{in}\;F\}\\ /⁎\mathrm{Calculating}\;\mathrm{the}\;\mathrm{insertion}\;\mathrm{position}\;\mathrm{in}\;\mathrm{the}\;\mathrm{public}\;\mathrm{model}\;\mathrm{of}\;\mathrm{the}\;\mathrm{change}\;\mathrm{initiator}⁎/\\ \mathrm{if}\;\psi (\mathrm{pred})\neq \emptyset \;\mathrm{then}\\ \left|\;\mathrm{pred}\prime \leftarrow \psi (\mathrm{pred}\right)\\ \mathrm{else}\\ \left|\;\mathrm{pred}\prime \leftarrow \{\psi (n)/n\in T\_{\mathrm{preset}}_{\left(\mathrm{interaction}\right)}(\mathrm{pred})\right\}\\ \mathrm{end}\\ \mathrm{if}\;\psi (\mathrm{succ})\neq \emptyset \;\mathrm{then}\\ \left|\mathrm{succ}\prime \leftarrow \psi (\mathrm{succ}\right)\\ \mathrm{else}\\ \left|\mathrm{succ}\prime \leftarrow \{\psi (n)/n\in T\_{\mathrm{postset}}_{\left(\mathrm{interaction}\right)}(\mathrm{succ}\right)\\ \mathrm{end}\\ \Delta \leftarrow \Delta \wedge (\mathrm{Insert}(\psi ○{\mathrm{abstr}}_{\left(\mathrm{interaction}\right)}(F),\mathrm{pred}\prime ,\mathrm{succ}\prime ),l_{p})\\ /⁎\mathrm{Calculating}\;\mathrm{the}\;\mathrm{insertion}\;\mathrm{position}\;\mathrm{in}\;\mathrm{the}\;\mathrm{public}\;\mathrm{model}\;\mathrm{of}\;\mathrm{each}\;\mathrm{partner}\;\mathrm{involved}\;\mathrm{in}\;\mathrm{the}\;\mathrm{change}⁎/\\ \mathrm{for}\;\mathrm{each}\;p\prime \in P_{\Delta }\;\mathrm{do}\\ |\begin{array}{c} \mathrm{for}\;\mathrm{each}\;n\in \mathrm{pred}\prime \;\mathrm{do}\\ |\begin{array}{c} \mathrm{if}\;\varphi (n)\neq \emptyset \;\mathrm{then}\\ \left|\mathrm{pred}\prime \prime \leftarrow \mathrm{pred}\prime \prime \cup \{\xi (n)\right\}\\ \mathrm{else}\\ \left|\mathrm{pred}\prime \prime \leftarrow \mathrm{pred}\prime \prime \cup \{\xi (n\prime )/n\prime \in T\_{\mathrm{preset}}_{p\prime }(n)\right\}\\ \mathrm{end} \end{array}\\ \mathrm{end}\\ \mathrm{for}\;\mathrm{each}\;n\in \mathrm{succ}\prime \;\mathrm{do}\\ |\begin{array}{c} \mathrm{if}\;\varphi (n)\neq \emptyset \;\mathrm{then}\\ \left|\mathrm{succ}\prime \prime \leftarrow \mathrm{succ}\prime \prime \cup \{\xi (n)\right\}\\ \mathrm{else}\\ \left|\mathrm{succ}\prime \prime \leftarrow \mathrm{succ}\prime \prime \cup \{\xi (n\prime )/n\prime \in T\_{\mathrm{postset}}_{p\prime }(n)\right\}\\ \mathrm{end} \end{array}\\ \mathrm{end}\\ F_{p\prime }\leftarrow \varphi ○{\mathrm{abstr}}_{p\prime }○\psi ○{\mathrm{abstr}}_{\left(\mathrm{interaction}\right)}(F)\\ \mathrm{posIn}\leftarrow \xi (\mathrm{pred}\prime \prime )\\ \left|\mathrm{posOut}\leftarrow \xi (\mathrm{succ}\prime \prime \right)\\ \mathrm{if}(\exists n\in L_{p\prime }\;\mathrm{between}\;\mathrm{posIn}\;\mathrm{and}\;\mathrm{posOut})\;\mathrm{then}\\ \left|\Delta \leftarrow \Delta \wedge (\mathrm{Insert}({\overset{¯}{F}}_{p\prime },\mathrm{Parallel},\mathrm{posIn},\mathrm{posOut}),l_{p}\right)\\ \mathrm{else}\\ \left|\Delta \leftarrow \Delta \wedge (\mathrm{Insert}({\overset{¯}{F}}_{p\prime },\mathrm{Sequence},\mathrm{posIn},\mathrm{posOut}),l_{p}\right)\\ \mathrm{end} \end{array}\\ \mathrm{end} \end{array}\\ \mathrm{end}\\ \mathrm{Output}:\Delta /⁎\mathrm{List}\;\mathrm{of}\;\mathrm{changes}\;\mathrm{to}\;\mathrm{be}\;\mathrm{propagated}⁎/ \end{array}$
**49end**

## Appendix A Propagation of fragment deletions

This appendix describes Algorithm 1, which shows the steps required to determine the effects of a change request of type DELETE has on a choreography.

1. Isolated or propagating changes (*cf. Lines* 4–5 *in* Algorithm 1).
2. Private-to-Public effects (*cf. Line* 9 *in* Algorithm 1).
3. Public-to-Public effects (*cf. Lines* 10–16 *in* Algorithm 1).

Algorithm 1 Delete operation propagation: $\mathrm{Delete}\_{\mathrm{Propag}}_{\pi_{p}}(F)$.

## Appendix B Propagation of fragment replacements

This appendix describes Algorithm 2, which shows the steps to determine the propagation effects of a fragment replacement in choreographies.

1. Isolated or propagating changes (*cf. Lines* 7–8 *in* Algorithm 2).
2. Private-to-Public effects (*cf. Lines* 10–11 *in* Algorithm 2).
3. Public-to-Public effects (*cf. Lines* 12–33 *in* Algorithm 2).

Algorithm 2 Replace operation propagation: $\mathrm{Replace}\_{\mathrm{Propag}}_{\pi_{p}}(F,\;F\prime )$.

## Appendix C Propagation of fragment insertions

In this appendix, Algorithm 3 is presented, which summarizes the steps required for propagating a change operation of type INSERT to a particular process partner.

1. Isolated or propagating changes(*cf. Lines* 5–6 *in* Algorithm 3):
2. Private-to-Public effects (*cf. Lines* 11–20 *in* Algorithm 3):
3. Public-to-Public effects (*cf. Lines* 23–46 *in* Algorithm 3):

Algorithm 3 Insert operation propagation: $\mathrm{Insert}\_{\mathrm{Propag}}_{\pi_{p}}(F,\mathrm{pred},\mathrm{succ})$.

## Footnotes

1 www.aristaflow.com

2 cpee.org

3 We use the type definition syntax of the ML language [19].

4 https://code.google.com/p/jbpt/

## References

[1] Schulte S., Schuller D., Steinmetz R., Abels S. (2012). Plug-and-play virtual factories. IEEE Internet Comput..

[2] F.M. Besson, P.M. Leal, F. Kon, Towards Verification and Validation of Choreographies. Technical Report, Department of Computer Science, University of Sao Paulo, 2011.

[3] Peltz C. (2003). Web services orchestration and choreography. Computer.

[4] W. van der Aalst, A decade of business process management conferences: personal reflections on a developing discipline, in: International Conference on Business Process Management, 2012, pp. 1–16.

[5] Casati F., Ceri S., Pernici B., Pozzi G. (1998). Workflow evolution. Data Knowl. Eng..

[6] Rinderle S., Reichert M., Dadam P. (2004). Correctness criteria for dynamic changes in workflow systems: a survey. Data Knowl. Eng..

[7] R. Breu, et al., Towards living inter-organizational processes, in: IEEE International Conference on Business Informatics, 2013, pp. 363–366.

[8] W. Fdhila, S. Rinderle-Ma, M. Reichert, Change propagation in collaborative processes scenarios, in: International Conference on Collaborative Computing: Networking, Applications and Worksharing, 2012, pp. 452–461.

[9] S. Rinderle, A. Wombacher, M. Reichert, Evolution of process choreographies in DYCHOR, in: International Conference on Cooperative Information Systems, 2006, pp. 273–290.

[10] W. Fdhila, S. Rinderle-Ma, Predicting change propagation impacts in collaborative business processes, in: ACM Symposium on Applied Computing (SAC), 2014, pp. 1378–1385.

[11] W. Fdhila, S. Rinderle-Ma, C. Indiono, Memetic algorithms for mining change logs in process choreographies, in: International Conference on Service-Oriented Computing (ICSOC), 2014, pp. 47–62.

[12] Vanhatalo J., Völzer H., Koehler J. (2008). The refined process structure tree. Bus. Process Manag..

[13] Weber B., Reichert M., Rinderle-Ma S. (2008). Change patterns and change support features—enhancing flexibility in process-aware information systems. Data Knowl. Eng..

[14] Signavio: Signavio process editor. 〈http://academic.signavio.com/〉Accessed 2013-10-24.

[15] G. Decker, M. Weske, Behavioral consistency for B2B process integration, in: International Conference on Advanced Information Systems Engineering, 2007, pp. 81–95.

[16] Barros A., Dumas M., Oaks P. (2006). Standards for web service choreography and orchestration: status and perspectives. Bus. Process Manag. Workshops.

[17] C.C. Ekanayake, M. Dumas, L. García-Bañuelos, M.L. Rosa, Slice, mine and dice: complexity-aware automated discovery of business process models, in: International Conference on Business Process Management, 2013, pp. 49–64.

[18] Polyvyanyy A., Garcia-Banuelos L., Dumas M. (2012). Structuring acyclic process models. Inf. Syst..

[19] Milner R., Tofte M., Macqueen D. (1997). The Definition of Standard ML.

[20] B. van Dongen, W. van der Aalst, H. Verbeek, Verification of EPCs: using reduction rules and petri nets, in: International Conference on Advanced Information Systems Engineering, 2005, pp. 372–386.

[21] W. Fdhila, U. Yildiz, C. Godart, A flexible approach for automatic process decentralization using dependency tables, in: International Conference on Web Services, 2009, pp. 847–855.

[22] M.L. Rosa, M. Dumas, R. Uba, R.M. Dijkman, Business process model merging: an approach to business process consolidation, in: ACM Transactions on Software Engineering and Methodology, 2012.

[23] Gottschalk F., van der Aalst W., Jansen-Vullers M.H. (2008). Merging event-driven process chains. On the Move to Meaningful Internet Systems.

[24] F. Puhlmann, M. Weske, Interaction soundness for service orchestrations, in: International Conference on Service-Oriented Computing, 2006, pp. 302–313.

[25] W. Fdhila, M. Rouached, C. Godart, Communications semantics for WSBPEL processes, in: International Conference on Web Services, 2008, pp. 185–194.

[26] H. Foster, S. Uchitel, J. Magee, J. Kramer, Compatibility verification for web service choreography, in: International Conference on Web Services, 2004, pp. 738–741.

[27] Rouached M., Fdhila W., Godart C. (2010). Web services compositions modelling and choreographies analysis. Int. J. Web Service Res..

[28] Rouached M., Fdhila W., Godart C. (2009). A semantical framework to engineering WSBPEL processes. Inf. Syst. e-Bus. Manag..

[29] W. van der Aalst, M. Weske, The P2P approach to interorganizational workflows, in: International Conference on Advanced Information Systems Engineering, 2001, pp. 140–156.

[30] van der Aalst W., Lohmann N., Massuthe P., Stahl C., Wolf K. (2008). From public views to private views—correctness-by-design for services. Web Services Formal Methods.

[31] Bohner S.A., Arnold R.S. (1996). Software Change Impact Analysis.

[32] Giffin M., de Weck O., Bounova G., Keller R., Eckert C., Clarkson P.J. (2009). Change propagation analysis in complex technical systems. J. Mech. Des..

[33] G.A. Oliva, G. de Maio Nogueira, L.F. Leite, M.A. Gerosa, Choreography Dynamic Adaptation Prototype, Technical Report, Universidade de Sao Paulo, 2012.

[34] Clarkson P.J., Simons C., Eckert C. (2004). Predicting change propagation in complex design. J. Mech. Des..

[35] B. Weber, S. Rinderle-Ma, M. Reichert, Change patterns and change support features in process-aware information systems, in: International Conference on Advanced Information Systems Engineering, 2007, pp. 574–588.

[36] L. Steffen, A Review of Software Change Impact Analysis, Technical Report, Universitätsbibliothek Ilmenau, 2011.

[37] Eckert C.M., Keller R., Earl C., Clarkson P.J. (2006). Supporting change processes in design: complexity, prediction and reliability. Reliab. Eng. Syst. Saf..

[38] Eckert C., Zanker W., Clarkson P.J. (2001).

[39] van der Aalst W., Basten T. (2002). Inheritance of workflows: an approach to tackling problems related to change. Theor. Comput. Sci..

[40] W. Fdhila, A. Baouab, K. Dahman, C. Godart, O. Perrin, F. Charoy, Change propagation in decentralized composite web services, in: International Conference on Collaborative Computing, 2011, pp. 508–511.

[41] W. Fdhila, S. Rinderle-Ma, A. Baouab, O. Perrin, C. Godart, On evolving partitioned web service orchestrations, in: International Conference on Service-Oriented Computing and Applications, 2012, pp. 1–6.

[42] M. Reichert, T. Bauer, Supporting ad-hoc changes in distributed workflow management systems, in: International Conference on Cooperative Information Systems, 2007, pp. 150–168.

[43] Hens P., Snoeck M., De Backer M., Poels G. (2014). Verification of change in a fragmented event-based process coordination environment. IEEE Trans. Services Comput..

[44] Küster J.M., Gerth C., Engels G. (2010). Dynamic computation of change operations in version management of business process models. Model. Found. Appl..

[45] Atluri V., Chun S.A. (2003). Handling dynamic changes in decentralized workflow execution environments. Database Expert Syst. Appl..

[46] Weidlich M., Mendling J., Weske M. (2012). Propagating changes between aligned process models. J. Syst. Softw..

[47] K.H. Dam, M. Winikoff, Cost-based BDI plan selection for change propagation, in: International Joint Conference on Autonomous Agents and Multiagent Systems, 2008, pp. 217–224.

[48] H.K. Dam, A. Ghose, Supporting change propagation in the maintenance and evolution of service-oriented architectures, in: Asia Pacific Software Engineering Conference, 2010, pp. 156–165.

[49] Mafazi S., Grossmann G., Mayer W., Stumptner M. (2013). On-the-fly change propagation for the co-evolution of business processes. On the Move to Meaningful Internet Systems.

[50] J. Kolb, K. Kammerer, M. Reichert, Updatable process views for user-centered adaption of large process models, in: International Conference on Service Oriented Computing, 2012, pp. 484–498.

[51] A. Mahfouz, L. Barroca, R. Laney, B. Nuseibeh, Requirements-driven collaborative choreography customization, in: International Conference on Service-Oriented Computing, 2009, pp. 144–158.

[52] Mallya A.U., Singh M.P. (2006). Incorporating commitment protocols into tropos. Agent-Oriented Software Engineering VI.

[53] K. Dahman, F. Charoy, C. Godart, Alignment and change propagation between business processes and service-oriented architectures, in: International Conference on Service Computing, 2013, pp. 168–175.

[54] Roohi N., Salaün G., France V. (2011). Realizability and dynamic reconfiguration of chor specifications. Informatica.

[55] D. Knuplesch, M. Reichert, R. Pryss, W. Fdhila, S. Rinderle-Ma, Ensuring compliance of distributed and collaborative workflows, in: International Conference on Collaborative Computing, 2013, pp. 133–142.

[56] D. Knuplesch, M. Reichert, W. Fdhila, S. Rinderle-Ma, On enabling compliance of cross-organizational business processes, in: International Conference on Business Process Management, 2013, pp. 146–154.

[57] S. Rinderle, M. Reichert, M. Jurisch, U. Kreher, On representing, purging, and utilizing change logs in process management systems, in: International Conference on Business Process Management, 2006, pp. 241–256.

[58] Lenz R., Reichert M. (2007). IT support for healthcare processes—premises, challenges, perspectives. Data Knowl. Eng..

[59] Paurobally S., Tamma V., Wooldrdige M. (2007). A framework for web service negotiation. ACM Trans. Auton. Adap. Syst..

[60] R. Vigne, J. Mangler, E. Schikuta, S. Rinderle-Ma, WS-agreement based service negotiation in a heterogeneous service environment, in: International Conference on Service-Oriented Computer and Applications, 2012, pp. 1–8.

[61] VDA Recommendation 4965 T1: Engineering Change Management (ECM)—Part 1: Engineering Change Request (ECR) Version 1.1, 2005.

[62] D. Müller, J. Herbst, M. Hammori, M. Reichert, IT support for release management processes in the automotive industry, in: International Conference on Business Process Management, 2006, pp. 368–377.

[63] B. Schultheiss, J. Meyer, R. Mangold, T. Zemmler, M. Reichert, Designing the Processes for Ovarian Cancer Surgery, Technical Report DBIS-6, University of Ulm, 1996.

